# Immune cell disorder in viral pneumonia

**DOI:** 10.1186/s12967-026-08193-z

**Published:** 2026-05-16

**Authors:** Yunfang Zhang, Fengsen Li, Ling Wang, Dan Xu, Jing Jing, Xiangpeng Li, Qianqian Liang, Zheng Li

**Affiliations:** 1 The Fourth Clinical Medical College of Xinjiang Medical University, Urumqi, Xinjiang 830000 China; 2 Xinjiang Uygur Autonomous Region Hospital of Traditional Chinese Medicine, Urumqi, Xinjiang 830000 China; 3 Xinjiang Key Laboratory of Respiratory Disease Research, Xinjiang Clinical Research Center for Respiratory System Diseases, Xinjiang National Clinical Research Base of Traditional Chinese Medicine, Urumqi, Xinjiang 830000 China

**Keywords:** Viral pneumonia, Immune cells, Review

## Abstract

In recent years, the complexity of viral infections has escalated, leading to a steady rise in the number of patients suffering from viral pneumonia. Both innate and adaptive immune cells play a crucial role in the development and clinical presentation of this condition. Viruses not only initiate the antiviral immune response within the human body but also provoke uncontrolled inflammatory reactions. Severe viral infections are often marked by a “cytokine storm,” characterized by an excessive release of proinflammatory cytokines. This cytokine storm can lead to abnormalities in immune cells, particularly neutrophils and macrophages, as well as dysfunction in lymphocytes. Such disruptions caused by respiratory viruses may result in respiratory failure, significant multi-organ dysfunction, and sepsis. Therefore, understanding the pathogenic mechanisms through which respiratory viral infections impact immune cells is crucial for guiding clinical treatment and prognosis. Furthermore, effectively modulating immune cell responses in virus-infected hosts—by enhancing antiviral immunity and mitigating systemic inflammation—may be essential for treating viral pneumonia. This review highlights the roles of various immune cells in viral pneumonia and explores the mechanisms by which viral infections induce immune cell abnormalities, offering insights for future clinical strategies and improving prognosis for respiratory viral infections.

**Supplementary Information:**

The online version contains supplementary material available at 10.1186/s12967-026-08193-z.

## Introduction

Community-acquired pneumonia (CAP) represents a significant health challenge both in China and worldwide [1]. It is one of the leading causes of mortality, responsible for approximately 2.56 million deaths annually [2]. Over the past two decades, three significant outbreaks of coronavirus-related pneumonia have occurred, including Severe acute respiratory syndrome(SARS), Middle east respiratory syndrome (MERS), and Corona virus disease-2019(COVID-19). The COVID-19 pandemic has notably altered the landscape of viral pneumonia, amplifying the focus on viruses as critical pathogens [3–5]. With advancements in detection technology and the introduction of novel diagnostic methods, it is now possible to identify a growing number of viruses simultaneously. This progress has led to an increase in diagnoses of viral pneumonia and a heightened recognition of viruses as significant contributors to both mild and severe respiratory infections [4]. Current estimates suggest that the probability of viral infection in immunocompetent patients with CAP ranges from 27.5% to 39.2% [1, 6]. The most prevalent respiratory viruses associated with viral pneumonia include the influenza virus, respiratory syncytial virus, adenovirus, and rhinovirus [5, 7–9]. The condition can deteriorate rapidly in elderly or immunocompromised individuals, leading to heightened mortality rates [4, 10].

**Fig. 1:**
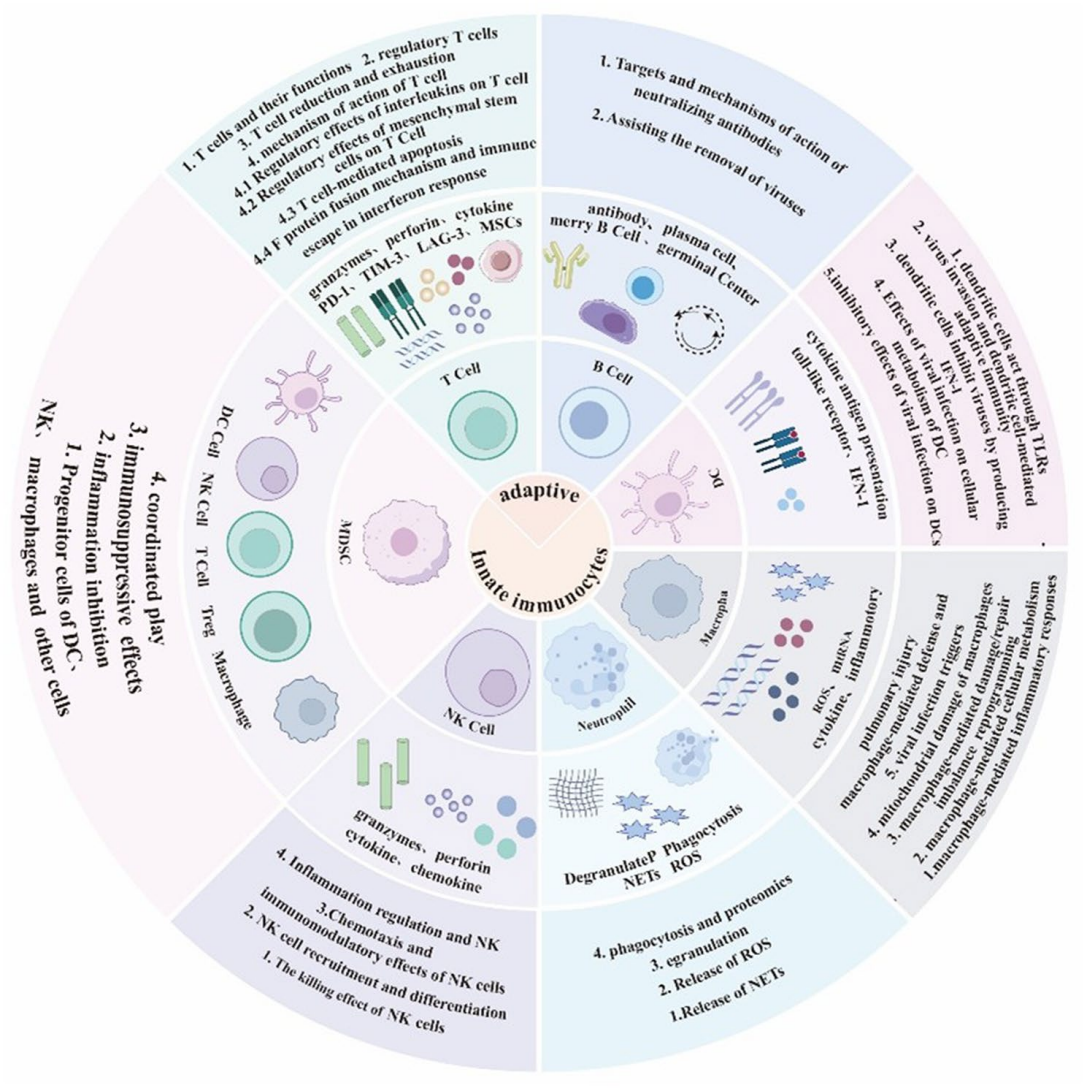
Summary diagram illustrating the mechanisms of immune cell responses during viral infection

Immune cells play a crucial role in the infection process of viral pneumonia. The functionality of these cells significantly influences the outcomes and prognosis of the disease. “Immune cell dysregulation” is not a single disease entity, but rather a collective term for a diverse range of pathological states. Based on the functional characteristics of immune cells in viral pneumonia, this review broadly defines “immune cell dysregulation” as three interrelated yet pathologically distinct abnormal states. (1) Excessive activation leads to cytokine storm [11]. During the acute phase of viral infection, particularly when macrophages and neutrophils overreact to TLR signaling, cytokine storms (characterized by the excessive expression and release of pro-inflammatory factors such as IL-6, TNF-α, and IFN-γ) and the excessive formation of neutrophil extracellular traps (NETs) occur [12, 13], exacerbating inflammatory responses and causing immune-mediated lung injury.Persistent inflammation can induce the transformation of NK cells into exhausted phenotypic NK cells [14], leading to rapid depletion of NK cells and further impairing their ability to effectively control inflammation. (2) Persistent suppression leads to immune exhaustion. After pathogen clearance, the immune system fails to promptly restore homeostasis and enters a sustained “suppression-exhaustion” state. For instance, T cells continuously overexpress inhibitory receptors such as PD-1 and TIM-3, which impair host immunity [14]; myeloid-derived suppressor cells abnormally proliferate by secreting Arg-1, ROS, and IL-10, suppressing the functions of T cells, NK cells, and dendritic cells (DCs), resulting in immunosuppression and exacerbating tissue damage [15–18]. (3) Functional defects lead to inadequate immune responses. Underlying conditions such as diabetes, tumors, aging, or primary immunodeficiency can impair the ability of immune cells to establish an effective antiviral response—This may mainfest as impaired dendritic cell (DC) migration and antigen presentation—which results in the failure to effectively activate initial T cells [19]; Abnormal macrophage polarization; failure of initial expansion of NK cells and T cells; and delayed or inhibited neutralizing antibody production. These factors collectively lead to ineffective initiation of antiviral responses during the early stages of infection, predisposing individuals to severe disease progression [20, 21] (Supplementary Table 1). In this study, we systematically elucidiated the mechanistic characteristics of functional changes in various immune cells during the pathogenesis of viral pneumonia, serving as a reference for future multi-pathway strategies aimed at mitigating pulmonary damage resulting from viral infections (Fig. 1).

## Innate immune cells

Human immune cells can be categorized into innate and adaptive types. Innate immune cells represent the first line of defense against infections caused by foreign pathogens, which primarily include neutrophils, NK cells, macrophages, and DC cells. Unlike adaptive immune cells, innate immune cells do not depend on antibody activation; rather, they can directly induce the expression of interferons through pathways involving pattern recognition receptors (PRRs). They release proinflammatory cytokines and chemokines to initiate immune responses against viruses, thereby limiting viral spread, recruiting immune cells to the sites of infection, enhancing defenses against viruses, and triggering the onset of adaptive immunity [22]. This process contributes significantly to disease control.

### NK cells

#### NK cells and their functions

**Fig. 2:**
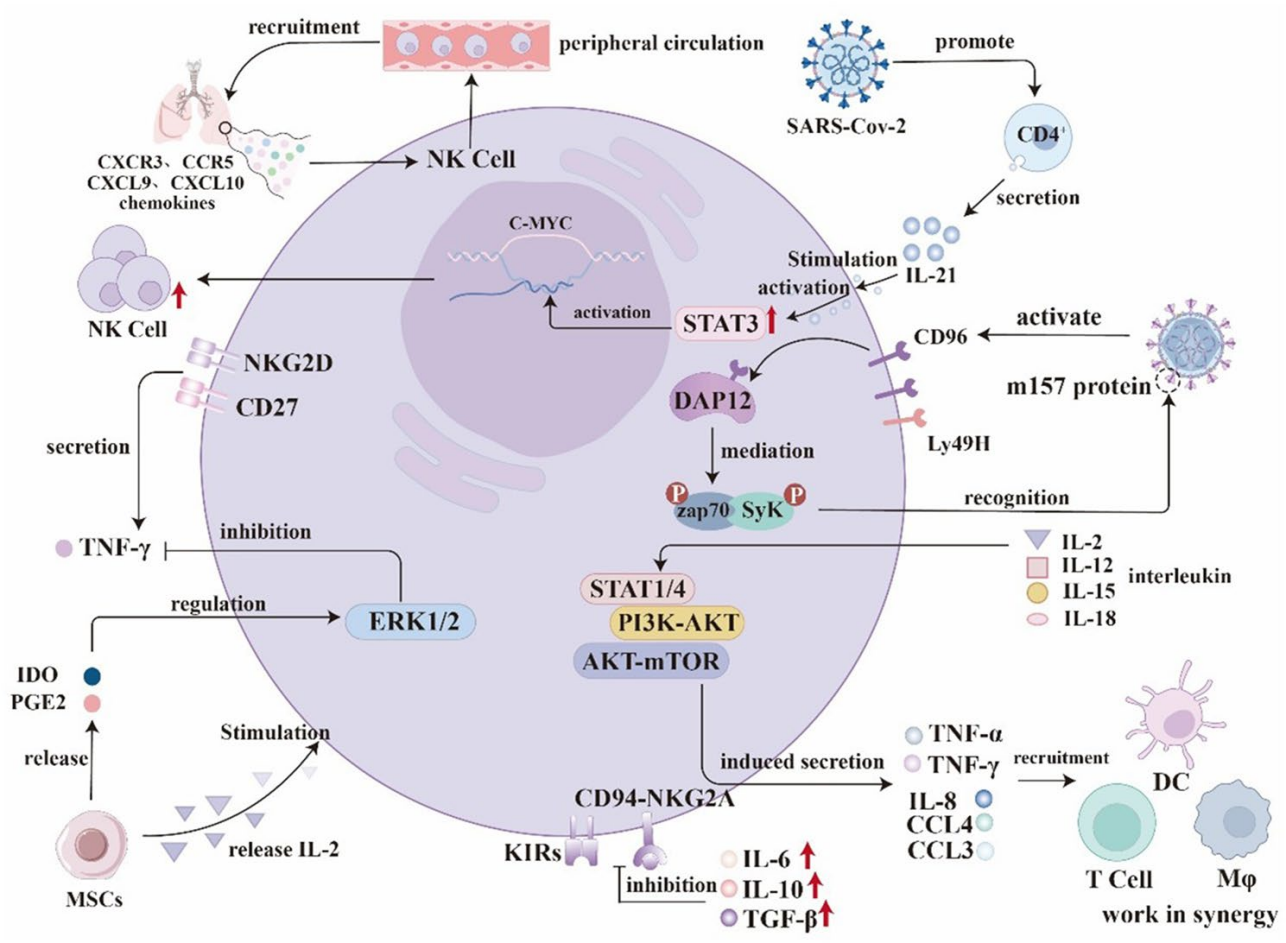
Mechanism of viral infection in NK cells

As a fundamental component of innate immunity, NK cells are essential for early defense against viral infections [23, 24]. They regulate immune responses by engaging in direct cytotoxic activities that involve granzymes and perforin. Additionally, they secrete cytokines and chemokines (e.g., tumor necrosis factor-α(TNF-α) and interferon-γ(IFN-γ)), which play crucial roles in shaping adaptive immunity and providing antiviral effects [25]. The cytotoxic capabilities of NK cells depend on their ability to form immune synapses with target cells [26]. Upon recognizing these target cells, NK cells establish lytic synapses and facilitate the release of perforin and cytotoxic granzymes to induce apoptosis in the targets [23] (Fig. 2, Supplementary Table 2).

NK cells are primarily categorized into two subgroups: the CD56^dim^CD16^high^ NK cells, which are abundant in peripheral circulation and exhibit strong cytotoxic capabilities, and the CD56^bright^CD16^low^ NK cells, found in tissues and lymphoid organs, which are associated with cytokine production [23, 25, 27]. These cells possess numerous inhibitory and activating receptors. The Major histocompatibility complex (MHC) class I serves as the key recognition element for inhibitory receptors, allowing NK cells to identify self-cells. When infected with SARS-CoV-2 and CMV viruses, infected cells often lose MHC class I expression, preventing interaction with NK cell inhibitory receptors. Consequently, this absence leads to NK cell activation, prompting the release of cytotoxic agents such as perforin and granzymes, or inducing apoptosis through death receptors like TNF-related apoptosis-inducing ligand (TRAIL) and Factor related apoptosis ligand (FasL) [23, 28]. Beyond their direct cytotoxic effects, NK cells are crucial early producers of IFN-γ during antiviral immune responses, which inhibits the replication of vaccinia virus (VACA) in the lungs and helps to contain the spread of viruses to other tissues [29]. Research indicates that a proinflammatory cytokine storm significantly contributes to lung injury and mortality during respiratory viral infections. IFN-γ, a pivotal component of this cytokine storm, is produced in large quantities by NK cells in response to RSV virus infection. This overproduction of IFN-γ exacerbates the cytokine storm, leading to heightened immune-mediated damage to lung tissue [30].

#### The role and mechanism of NK cells in viral infection

① The Role of NK Cells in Viral Infection. During the initial phase of RSV virus infection, NK cells, which are crucial innate immune cells in the lungs, accumulate and become activated. These activated NK cells exhibit elevated levels of Natural killer group 2(NKG2D) and CD27 and release significant amounts of IFN-γ, which damages virus-infected cells and contributes to acute pulmonary immune injury in the early stages of infection [30]. However, increased levels of cytokines—such as interleukin (IL-)-6, IL-10, and transforming growth factor (TGF-β)—produced by SARS-CoV-2 virus-infected cells or activated antigen-presenting cells (APCs) can inhibit NK cell activation. This occurs through the engagement of immunoglobulin-like receptors (KIRs) and the C-type lectin-like receptor CD94-NKG2A, which decreases the production of IFN-γ and granzyme B. As a result, the capacity of NK cells to eliminate infected cells is compromised, leading to a worsening of the infection [28, 31]. Additionally, CD96, an immune receptor expressed on NK cells, can function as an activating receptor during CMV virus infection. The activated NK receptor Ly49H transmits activation signals via DAP12, facilitating the phosphorylation of tyrosine kinases Zap70 and Syk. This receptor specifically recognizes the m157 protein on the surface of giant cells infected with viruses, thereby enhancing the anti-viral activity against these cells [32].

② NK cell recruitment and differentiation. The reduction of NK cells in peripheral blood correlates with the migration of activated NK cells from the bloodstream to lung tissue, playing a role in the pathogenesis of IV virus pneumonia [33]. NK cells are among the first responders to IAV virus infection. In the lungs of infected patients, elevated levels of chemokines—such as chemokine C-X-C motif receptor 3(CXCR3), chemokine C-C motif receptor 5(CCR5), chemokine C-X-C motif ligand (CXCL)9, CXCL10, and CXCL11—facilitate the recruitment of CD56^bright^ and CD56^dim^ NK cells from peripheral circulation into the lungs [34]. Infection with SARS-CoV-2 prompts activated CD4^+^T cells to produce IL-21 [35], which in turn stimulates NK cells and induces a high expression of signal transducer and activator of transcription 3(STAT3). This activation leads to downstream effects on c-Myc, ultimately promoting NK cell proliferation [36].

③ Chemotaxis and Immunomodulatory Functions of NK Cells. Soluble mediators generated during viral infections, such as interleukin (IL)-2, -12, -15 and − 18 [23], can stimulate NK cells to produce cytokines (e.g., TNF-α and IFN-γ) and chemokines (C-C motif chemokine ligand (CCL)3, CCL4 and IL-8) through the activation of signaling pathways like signal transducer and transcription activator 1(STAT1), signal transducer and activator of transcription 4(STAT4), and the PI3K-AKT-mTOR pathway. This process enhances the recruitment of other immune effector cells, thereby boosting the adaptive immune response [28, 34]. For example, IFN-γ produced by NK cells can promote the maturation and activation of dendritic cells, macrophages, and T cells, thereby regulating the immune responses of various immune cells [30, 37].

④ Inflammation Regulation and NK cells. The cytokine storm induced by viral infections represents an uncontrollable inflammatory response from the immune system [38]. Mesenchymal stem cells (MSCs) exhibit regulatory properties that play a crucial role in maintaining immune homeostasis, particularly during instances of overly activated or insufficiently activated immune responses [39]. Consequently, during viral infections, MSCs can modulate the function of NK cells and diminish the secretion of IFN-γ from NK cells stimulated by IL-2. Furthermore, MSCs can inhibit the proliferation of NK cells in an IFN-γ-dependent manner. They release indoleamine 2, 3-dioxygenase (IDO), prostaglandin E2(PGE2), and other factors that impede NK cell cytotoxicity by modulating the ERK1/2 signaling pathway and the expression of activation markers [40].

#### Macrophages

**Fig. 3:**
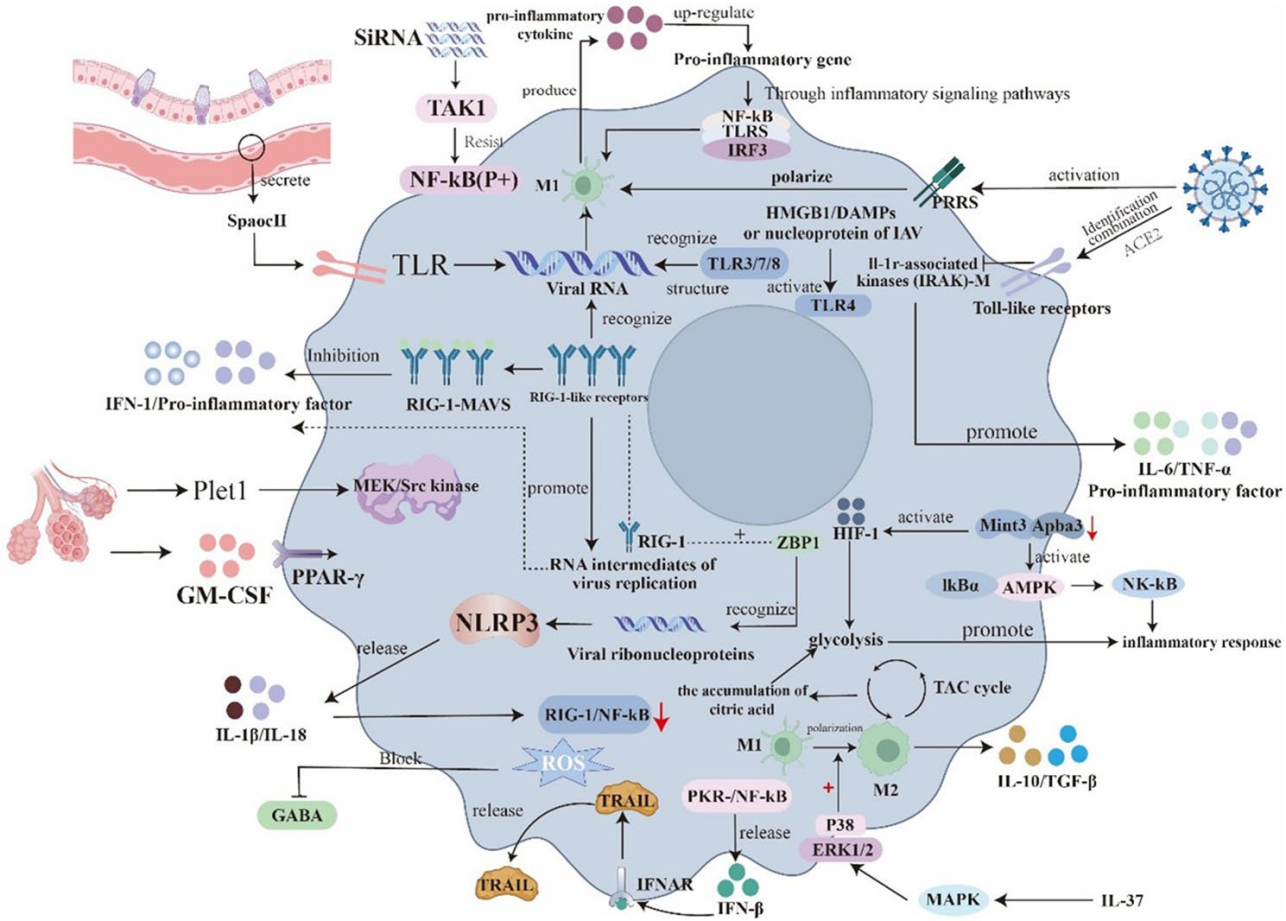
Mechanism of viral infection in macrophages

Alveolar macrophages, the predominant type of immune cells in the respiratory tract, serve as the first line of defense in innate immunity against viral infections [41–43]. These macrophages produce inflammatory cytokines that quickly identify and engulf virally infected cells, thereby curbing IAV viral replication. By releasing a range of cytokines and chemokines, they regulate local inflammatory responses and initiate subsequent adaptive immune responses. Alveolar macrophages also play a crucial role in recruiting peripheral immune cells to sites of infection, thereby mediating the innate immune response and significantly influencing the severity of viral infections [43–45]. Additionally, the recognition of pathogen associated molecular patterns (PAMPs) and by viruses, along with IFN-I signal transduction, can result in the establishment of innate immunological memory within alveolar macrophages, enhancing the efficacy of secondary antiviral responses [45] (Fig. 3, Supplementary Table 3).

#### Macrophage-mediated inflammatory responses

In SARS-CoV-2 viral infections, the activation of PRRs and the subsequent proinflammatory microenvironment drive macrophages towards the M1 phenotype. This shift leads to, the excessive production of proinflammatory cytokines, resulting in a phenomenon known as a cytokine storm. This heightened inflammatory response further amplifies the expression of inflammation-related genes, such as TNF, IL-6, IL-10, and Toll-like receptor (TLR)4. These genes modulate inflammation signaling pathways—specifically nuclear factor kappa B (NF-κB), TLRs, interferon regulatory factor 3(IRF3), and NOD-like receptor pyrin domain containing 3(NLRP3), leading to prolonged M1 macrophage activation and ongoing synthesis of inflammatory mediators [46]. In the context of COVID-19, SARS-CoV-2 binds to the angiotensin-converting enzyme 2(ACE2) receptor, triggering macrophages to hyperreact to TLRs. This interaction inhibits IL-1r-associated kinases (IRAK)-M, a key regulator of inflammatory responses, and promotes the overproduction of proinflammatory cytokines like IL-6 and TNF-α, which culminates in a cytokine storm and extensive pulmonary inflammation [47]. RIG-I-like receptors serve as intracellular sensors that detect RNA intermediates produced during viral replication, leading to the expression of IFN-I and other proinflammatory cytokines. In the context of infection caused by SARS-CoV-2 or IAV virus, alveolar macrophages activate the RIG-I-MAVS complex to produce IFN-I, enhancing the recruitment of inflammatory monocytes and restricting viral replication [43, 48]. Additionally, macrophages can identify RNA structures in phagocytosed virus-infected cells through TLR3, TLR7 and TLR8. TLR4 can also be activated by high mobility group box 1(HMGB1), damage-associated molecular patterns (DAMPs) or influenza virus A (IAV), triggering downstream proinflammatory or antiviral gene expression via the NF-κB or IRF3/7 signaling pathways. Furthermore, Z-DNA binding protein 1 (ZBP1) and RIG-I can detect viral ribonucleoproteins (vRNPs), activating the NLRP3 inflammasome, which results in the release of IL-1β and IL-18. Consequently, targeting and inhibiting downstream pathways such as RIG-I and NF-κB can mitigate the inflammatory responses in IAV virus-infected cells [43, 49]. In the context of SARS-CoV-2 infection, alveolar macrophages react to T cells by producing chemokines that draw T cells into the site of infection. Subsequently, T cells activate alveolar macrophages to release inflammatory cytokines through the production of IFN-γ. This interaction establishes a positive feedback loop that perpetuates the inflammatory responses [50].

#### Macrophage-mediated cellular metabolism reprogramming

Viral infections can lead to significant alterations in standard metabolic pathways, such as glycolysis, lipid metabolism, and amino acid metabolism, which are crucial for metabolic reprogramming in macrophages. Disruptions in the balance of key intermediates within these pathways, like spermidine and citric acid salts, can exacerbate viral diseases [51]. For example, the activation of the Wnt-β-catenin signaling pathway during influenza infection can result in the generation of proinflammatory macrophages, subsequently inducing the expression of proinflammatory cytokines, such as hypoxia-inducible factor (HIF-)-1α. HIF-1α promotes glycolytic metabolism, shifting the metabolic balance towards glycolysis and intensifying inflammation [52]. Influenza infection also enhances glycolysis for ATP production in macrophages by activating HIF-1 through Mint3/Apba3. The deletion of Mint3 leads to defects in glycolysis-induced ATP production, which can decrease the incidence of fatal influenza pneumonia. This reduction is linked to the weakening of the NF-κB signaling pathway, resulting in downregulation of cytokines and chemokines due to the upregulation of IκBα and activation of adenosine monophosphate-activated protein kinase (AMPK) [53]. In the case of COVID-19, the tricarboxylic acid cycle in M1 macrophages is inhibited, leading to the accumulation of citric acid. This accumulation serves as a substrate for the inflammatory functions of M1 macrophages, accelerating glycolytic metabolism, promoting inflammatory responses, and exacerbating pulmonary injury [54].

#### Macrophage-mediated damage/repair imbalance

In the absence of viral infection, alveolar macrophages remain relatively inactive. However, upon encountering a viral infection, these macrophages undergo polarization to the M1 phenotype, resulting in the production of proinflammatory cytokines such as IL-6, TNF-α and IL-1β, which drive inflammatory responses. Subsequently, M2 macrophages express IL-10 and TGF-β, playing a crucial role in pathogen clearance, inflammation inhibition, and tissue healing. If M1 macrophages remain active for an extended period, they can cause immunopathological damage and impede tissue recovery. Thus, the balance between M1 and M2 macrophages is essential for mediating the interplay between inflammatory damage and repair in the body [12, 43, 55]. During IV or SARS-CoV-2 virus infection, pulmonary capillary endothelial cells secrete Sparcl1, a stromal cell protein that promotes the polarization of macrophages to the M1 state via TLR4. This process exacerbates inflammation and intensifies the severity of viral pneumonia [13]. Additionally, placenta-expressed transcript 1 (Plet1) produced by alveolar macrophages has been shown to mitigate pulmonary injury caused by influenza virus infection. It achieves this by facilitating the repair of alveolar epithelial cells after viral pneumonia through the MEK and Src kinase signaling pathways [56]. IL-37, a novel member of the IL-1 family, is capable of shifting the cytokine balance during excessive inflammation. It encourages the polarization of macrophages from the M1 to the M2 phenotype through the Mitogen-activated protein kinase (MAPK) pathway, particularly via the ERK1/2 and p38 pathways. This shift leads to a reduction in inflammatory cytokine production, alleviating pulmonary injury caused by the influenza virus and promoting tissue repair [57]. Furthermore, the application of siRNA targeting Transforming growth factor-beta-activated kinase 1(TAK1) has been shown to significantly reduce pulmonary injury induced by RSV viral pneumonia. This effect is achieved by inhibiting the phosphorylation and activation of NF-κB, which subsequently decreases the release of proinflammatory cytokines from M1 macrophages [55].

#### Mitochondrial damage of macrophages

Mitochondria serve as vital signaling centers that regulate metabolism, apoptosis, antiviral immunity, and inflammatory responses. The preservation of mitochondrial integrity is essential for the survival and functionality of lung macrophages [42]. SARS-CoV-2 infection can disrupt mitochondrial function, which triggers the activation of proinflammatory M1 macrophages, exacerbating inflammatory responses and leading to pathological tissue damage [46]. Notably, macrophages with shortened telomeres can undergo a senescence-like transformation following respiratory viral infections, displaying features such as mitochondrial distress and aberrant activation of the STING and NLRP3 inflammasome pathways. This transformation enhances inflammatory responses and worsens immunopathology, potentially turning mild infections into severe viral pneumonia [58]. IAV viral infections induce mitochondrial disorganization, swelling, and the loss or fragmentation of cristae in macrophages through the gamma-aminobutyric acid (GABA) signaling pathway. Consequently, there is a decrease in mitochondrial DNA (mtDNA) levels and genes associated with mitochondrial respiratory complexes (I-V). Additionally, the expression of crucial molecules involved in mtDNA replication and mitochondrial production, such as Sirtuin 1(SIRT1), Peroxisome proliferator-activated receptor gamma coactivator 1 alpha (PGC-1α), and Transcription factor A (TFAM), is adversely affected. By inhibiting the GABA signaling pathway, it is possible to restore mitochondrial function, reduce oxidative stress and inflammation, and ultimately reestablish macrophage homeostasis in macrophages [42].

#### Viral infection triggers macrophage-mediated defense and pulmonary injury

In response to pathogen infections, granulocyte-macrophage colony-stimulating factor (GM-CSF) derived from alveolar epithelial cells stimulates alveolar macrophages to produce antimicrobial proteins, activate oxygen, and engage in phagocytosis to eliminate pathogens through Peroxisome proliferator activated receptor gamma (PPAR-γ) activation. Respiratory syncytial virus (RSV) infection enhances the expression of MHC II via interferon gamma receptor (IFNGR1) signaling, allowing IFN-γ to bolster the macrophage-dependent clearance of RSV in the lungs [48]. However, viral-infected alveolar macrophages also secrete IFN-β through the protein kinase R-(PKR-) and NF-κb signaling pathways. Through autocrine signalling via the macrophage type I interferon-α/β receptor (IFNAR), IFN-β stimulates the expression and release of TRAIL, which contributes to alveolar epithelial cell (AEC) injury and subsequent lung damage [49]. Proteomic analyses of the lower respiratory tract in patients with viral pneumonia revealed reduced expression of fatty acid binding protein (FABP) and macrophage inhibitory protein (MIP-5) involved in immune regulation, was reduced. These two proteins have multiple functions, including macrophage regulation, which affects the role of macrophages as the first line of defense in innate anti-virus defense [59].

### Neutrophils

#### Neutrophils and their main functions

**Fig. 4:**
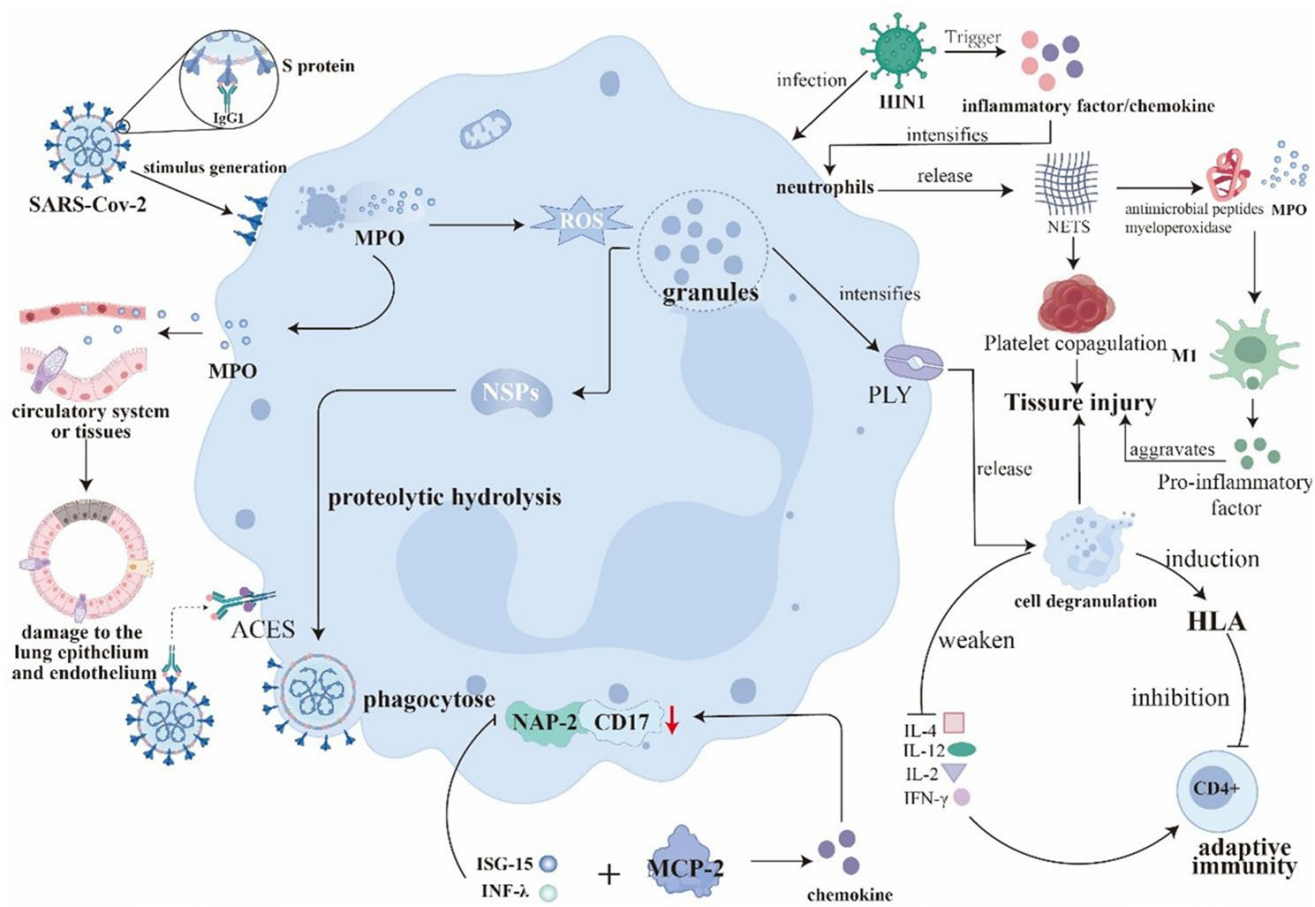
Mechanism of viral infection in neutrophils

Neutrophils are crucial innate immune cells capable of rapidly infiltrating affected tissues during infections or sterile injuries. In the context of viral infections, neutrophils eliminate pathogens through mechanisms such as phagocytosis, degranulation, and the release of reactive oxygen species (ROS) and neutrophil extracellular traps (NETs) [22, 60]. A moderate neutrophil response is protective against infections; however, excessive activation can lead to severe inflammation and tissue damage [61, 62]. The degree of neutrophil recruitment to the lungs correlates with the severity of disease caused by viral infections [60]. This recruitment is primarily mediated by significant increases in granulocyte colony-stimulating factor, chemokines, and proinflammatory cytokines (e.g., IL-6, INF-γ, tumor necrosis factor), which promote neutrophil activation and recruitment to inflamed lung tissue. Activated lymphocytes and neutrophils can alter the pathological state during recurrent infections, serving as key indicators of severe respiratory tract infections. Monitoring the counts of neutrophils and lymphocytes will reveal the disease progression. Studies confirmed that compared with patients with mild symptoms, patients requiring intensive care have significantly higher neutrophil counts and lower lymphocyte counts [63–66] (Fig. 4, Supplementary Table 4).

#### Release of NETs

NETs is a type of programmed cell death that serves a defensive role by capturing and killing pathogens [67]. Composed of extracellular DNA fibers along with histones and granule-derived proteins (e.g., elastase, myeloperoxidase), NETs are released by activated neutrophils, forming a trap for extracellular pathogens [12, 67–72]. During H1N1 infections, neutrophil recruitment is excessive and associated with elevated levels of inflammatory cytokines and chemokines, which promote NET formation [67]. As inflammation escalates, more neutrophils infiltrate the lung tissue and release substantial amounts of NETs. NETs’ antimicrobial peptides (LL-37) and myeloperoxidase (MPO) can polarize macrophages towards a proinflammatory (M1) phenotype, thereby exacerbating inflammatory responses and lung injury. Additionally, histones, a critical component of NETs, are highly toxic and can contribute to respiratory failure and systemic damage [12, 67–72]. NETs released into the extravascular space and circulation can also result in tissue damage and abnormal coagulation, significantly influencing the coagulation cascade. Reducing NET formation may alleviate inflammation and thrombosis associated with viral infections [68, 73, 74]. SARS-CoV-2 infection can activate NETs and directly induce *in vitroo* polymorphonuclear neutrophil (PMN) activation in vitro, leading to NET release and further progression of COVID-19 [75].

#### Release of ROS

The production of ROS is a critical signaling event in the progression of various inflammatory diseases, including respiratory viral infections. In patients with severe pneumonia, excessive neutrophil activation results in a robust ROS response, leading to oxidative stress. Oxidative stress and continued amplified systemic inflammation damage the capillary endothelium and thus destroy the thrombotic protection of ECs [76], leading to the progression of the disease. It has been demonstrated that, SARS-CoV-2 Omicron variant S protein and anti-spike proteins IgG1 can stimulate the release of MPO from PMNs. MPO, a key component of PMNs, produces highly reactive ROS and, when released into the extracellular space, can damage the lung epithelium and endothelium in COVID-19 patients, exacerbating oxidative lung tissue damage [75].

#### Degranulation

Studies of coronaviruses have underscored the significance of host proteases in viral replication and immune responses. Neutrophil serine proteases (NSPs), including cathepsin G (CatG), neutrophil elastase (NE) and protease 3 (PR3), are synthesized and stored in specialized lysosomal granules during early stages. The diverse array of antimicrobial substances housed in neutrophil granules is released into phagosomes to digest their contents or into the extracellular environment [77]. Neutrophil elastase, stored in granules and serving as a host defense factor, can cause neutrophil dissolution and leakage into the extracellular space under the influence of the pore-forming toxin pneumolysin (PLY). This process not only undermines the host’s innate immune response through proteolytic cleavage of TLRs and cytokines but also disrupts antigen presentation. The cleavage of human leukocyte antigen (HLA) molecules diminishes CD4^+^T cell activation and reduces cytokine levels, further exacerbating severe pneumonia associated with acute lung injury (ALI) and acute respiratory distress syndrome (ARDS). Additionally, neutrophil elastase can degrade various cytokines (e.g., INF-γ, IL-2, IL-4 and IL-12), compromising adaptive immunity [78, 79].

#### Phagocytosis and proteomics

As professional phagocytes, neutrophils directly engulf pathogens, infected or apoptotic cells, and other debris during infections, aiding host defense through their cytoplasmic hydrolases, nicotinamide adenine dinucleotide phosphate (NADPH) oxidases, and ROS production [60]. Proteomic analyses of viral lower respiratory tract infections indicated that patients with severe influenza virus or RSV infections exhibited elevated expression of interferon-related proteins (e.g., interferon-stimulated gene 15(ISG-15) and INF λ 1) and recombinant monocyte chemotactic protein 2(MCP-2), a chemokine induced by interferon signaling. Viral loads positively correlate with interferon signaling and negatively with neutrophil activation pathways. Type 1 interferon can inhibit neutrophil defenses, resulting in decreased expression of neutrophil-activating protein NAP-2 and neutrophil-activating glycoprotein CD177, ultimately impairing neutrophil antiviral efficacy [59]. In viral infections, the receptor binding domain (RBD) on the viral spike protein facilitates high-affinity interactions with the host cell receptor angiotensin-converting enzyme 2. promoting viral entry. However, NSPs can proteolytically cleave the viral spike protein, preventing viral entry and replication in vitro while altering subsequent IFN-induced CXCL10 production. NSPs can also directly inactivate cytokines, chemokines, and cell surface receptors, thereby mitigating inflammation and pulmonary injury caused by viral infections [77]. Compared to healthy controls and non-ICU patients, COVID-19 patients in the ICU show elevated levels of neutrophil granzyme proteins defensin alpha 3(DEFA3) and lipocalin-2(LCN2). Neutrophil serine proteases can activate and enhance the complement cascade on NETs, subsequently activating platelets and coagulation cascades. This overactivation of the immune system and coagulation dysfunction contributes to the severity of COVID-19 [80].

### Dendritic cells

#### Classification and function of dendritic cells

**Fig. 5:**
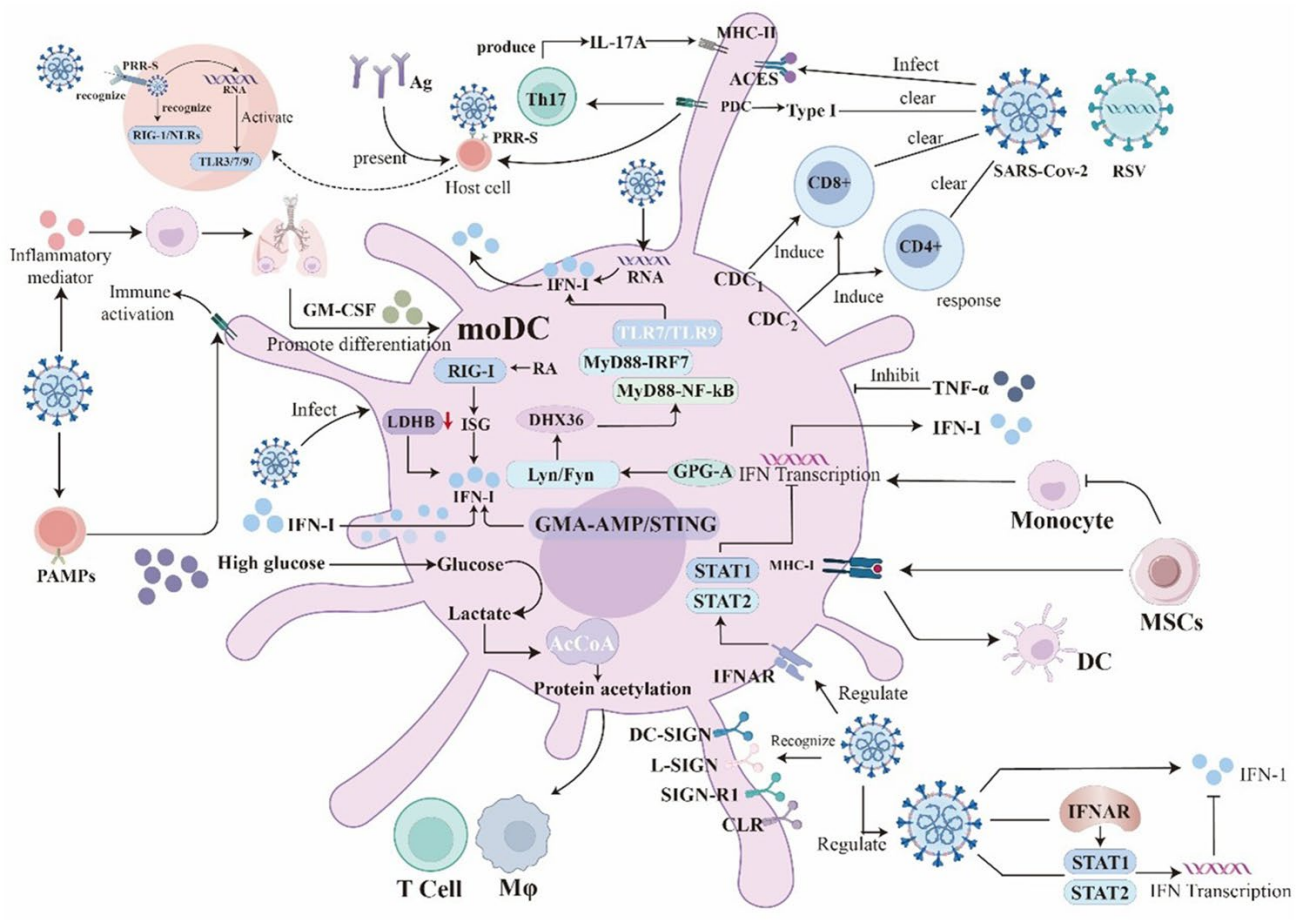
Mechanism of viral infection in DC cells

Dendritic cells are integral components of the immune system and function as specialized APCs that bridge innate and adaptive immunity [81–84], playing an important role in generating and sustaining antiviral immune responses [85]. DCs become activated through direct contact with pathogens or signals from macrophages and monocytes. Equipped with sensitive receptors, DCs can recognize PAMPs. Influenza virus invasion triggers these receptors, leading to the release of proinflammatory cytokines (e.g., IL-1, TNFα, IL-12), and anti-inflammatory cytokines (e.g., IL-4, IL-10, IFN-γ), thus initiating a comprehensive immune response with a focus on innate immunity [86]. During viral infections, proinflammatory cytokines facilitate the recruitment of monocytes from peripheral blood to the lungs. In the presence of GM-CSF, these monocytes differentiate into monocyte-derived DCs (moDCs) [87, 88]. Upon infection with viruses such as RSV or SARS-CoV-2, different DC subtypes exert complementary effects, inducing specific antiviral functions. Plasma cell-like dendritic cells (pDCs) produce type I interferons, while conventional DC1 (cDC1) promote virus-specific CD8 T cell responses, and conventional DC2 (cDC2) stimulate CD4 and CD8 T cell responses, ultimately facilitating viral clearance and alleviating symptoms [88, 89] (Fig. 5, Supplementary Table 5).

#### Dendritic cells act through TLRs

RSV virus can interact with PRRs on target cells, primarily belonging to the TLR family (TLR1/2/6 or TLR3). However, once inside the host cell, viral nucleic acids can activate PRRs located in endosomes, such as TLR3, TLR7, and TLR9. Moreover, viruses can also engage intracellular retinoic acid-inducible gene I (RIG-I)-like proteins (RLRs) (RIG-I, LGP2 and melanoma differentiation-related protein 5) and nod-like receptors (NLRs) (NLRC2, NLRP3 and NALP3) [88, 90]. Dendritic cells detect pathogens through PRRs such as TLRs, leading to the production of cytokines and chemokines while presenting antigens (Ag) to coordinate immune responses effectively [91]. Previous research has demonstrated that pDCs exhibit antiviral functions in response to TLRs stimulation [92]. pDCs can detect viral infections by internalizing nucleic acids and delivering them to endosomes containing TLRs, employing mechanisms such as clathrin-mediated endocytosis or autophagy [93]. For example, pDCs mediate the rapid production of type I (IFN-α/β, IFN-I) and type III (IL-28/29, IFN-λ, IFN-III) interferons and other cytokines like TNF-α, through the activation of TLR9 and TLR7. They can also internalize free virus particles and recognize viruses such as influenza A via endosomal TLRs in a replication-independent manner. Consequently, pDCs play a pivotal role in controlling both acute and persistent viral infections [91]. Gene expression patterns induced by TLR4 and TLR7/8 are highly similar, suggesting that TLR4 and other TLRs possess immunomodulatory functions in pDCs [92]. EB virus infections can also prompt T helper 17 (Th17) cells to produce proinflammatory cytokines like IL-17 A through TLR signaling, enhancing MHC II expression on DCs [93].

#### Virus invasion and dendritic cell-mediated adaptive immunity

Viruses can invade cells through angiotensin-converting enzyme 2, upregulating cell surface molecules (CD80, CD86 and MHC II) [84]. DCs and moDCs within tissues effectively activate adaptive immunity of CD8 and CD4 T cells through major MHC I and II molecules [87]. During influenza A viral infections, CCR2 chemokine receptor expression is upregulated, enabling DCs to respond to CCR2 ligands and promote targeted recruitment of CDCs to the infection site. Conversely, reduced CCR2 expression selectively impairs the expansion of cDC networks in the lungs of influenza A-infected individuals, weakening T cell responses to the virus [94]. IL-3 stimulates CXCL12 through epithelial cell activation, enhancing the recruitment of pDCs to the lungs during viral pneumonia and boosting antiviral immunity by enhancing pDC-mediated T cell activation in response to viral stimuli [88, 95].

#### Dendritic cells inhibit viruses by producing IFN-I

IFN-I is a crucial anti-viral cytokine, playing a vital role in limiting viral spread, restricting replication in infected cells, and bolstering both innate and adaptive immune responses against viruses. It is the core of protecting the host from viral infections. Deficiencies in the IFN-I response are associated with the severity of viral pneumonia. Dendritic cells, particularly those resembling plasma cells, are the primary source of IFN-I production and respond swiftly to viral infections by synthesizing and releasing substantial amounts of this cytokine [96–99]. During RSV virus infections, retinoic acid (RA) produced by dendritic cells enhances the expression of ISGs and facilitates IFN-I production, thereby regulating the RIG-I pathway to control the infection [100]. Fc receptors can facilitate the entry of immune complex nucleic acids into plasmacytoid pDCs, which triggers their production of IFN-I. TLR7 and TLR9, expressed by pDCs, activate the MyD88-NF-kB and MyD88-IRF7 signaling pathways to produce IFN-I. In pDCs, CpG-A is shuttled to early endosomes through Src kinases Lyn and Fyn, where it interacts with the cytoplasmic nucleic acid receptor DHX36, activating the MYD88-IRF7 pathway and inducing IFN-I production. pDCs also recognize pathogens via the cyclic GMP-AMP synthase (cGAS)-stimulator of STING pathway, leading to IFN-I expression. The mammalian target of rapamycin (mTOR) acts as a central regulator essential for IFN-I production in pDCs. IFN-I-driven cell apoptosis is also part of a negative feedback loop that limits pDC IFN-I production [89, 101]. Upon viral infection, the number of pDCs and the amount of IFN-I produced by pDCs are also related to the patient’s gender. Notably, specific genes involved in TLR signaling are located on the X chromosome, which may allow female pDCs with biallelic TLR7 genes to produce higher amounts of IFN-I, respond more rapidly to viral infections, and more effectively control viral replication compared to their male counterparts [88, 101].

#### Effects of viral infection on cellular metabolism of DC

Viral infections can significantly reduce the expression of the metabolic enzyme lactate dehydrogenase B (LDHB), which plays a crucial role as a positive regulator in the production of IFN-I by plasmacytoid pDCs. DCs utilize substantial amounts of lactate and glucose as carbon sources to facilitate oxidative metabolism. A deficiency in LDHB diminishes pDCs’ capacity to engage in both oxidative metabolism and glycolysis. This metabolic process is essential for pDCs to generate IFN-I, which is vital for curtailing viral replication and dissemination. Consequently, metabolic impairments following viral infection can hinder the antiviral capabilities of pDCs [96, 98, 101]. Furthermore, glucose serves as a crucial regulator of lung DC function during viral infections. Elevated glucose levels can shift metabolism away from lactate production towards the synthesis of acetyl-CoA. An overproduction of acetyl-CoA can disrupt metabolic pathways within DCs, leading to increased histone acetylation and affecting other immune cells, such as T cells and macrophages, thereby heightening susceptibility to viral infections [102]. IAV is detected by DCs through the recognition of PRRs that detect specific viral carbohydrate structures. Multiple CLRs expressed by dendritic cells, including DC-SIGN (CD209), L-SIGN (CD209L), and SIGN-R1, enhance the capability of DCs to sense the virus by binding to glycans found on IAV glycoproteins [89].

#### Inhibitory effects of viral infection on DCs

Virus-encoded structural proteins, non-structural protein (NSP) and auxiliary protein triads, can inhibit IFN transcription and prevent IFN response by modulating the IFNAR and its downstream signaling pathways, including STAT1 and STAT2 [103]. The non-structural protein 2 (NS2) of the influenza A virus specifically inhibits the maturation and antigen-presenting functions of dendritic cells. It achieves this by binding to exportin 5 (Xpo5) to block miRNA production while simultaneously interacting with IRF-3 [104]. Furthermore, TNFα, a well-known pro-inflammatory cytokine, can also suppress IFN-I production by pDCs [105]. Parabens have been shown to modulate antiviral immune responses by significantly decreasing the production of IFN-α1 in bone marrow-derived DCs (BMDCs) during viral infections [106]. MSCs prevent the differentiation of monocytes into DCs, reverse the maturation of DCs by downregulating MHC-II expression, and inhibit DC differentiation through the actions of IL-10 and direct cell-cell contact [40].

### Myeloid-derived suppressor cells (MDSCs)

#### MDSCs and their functions

**Fig. 6:**
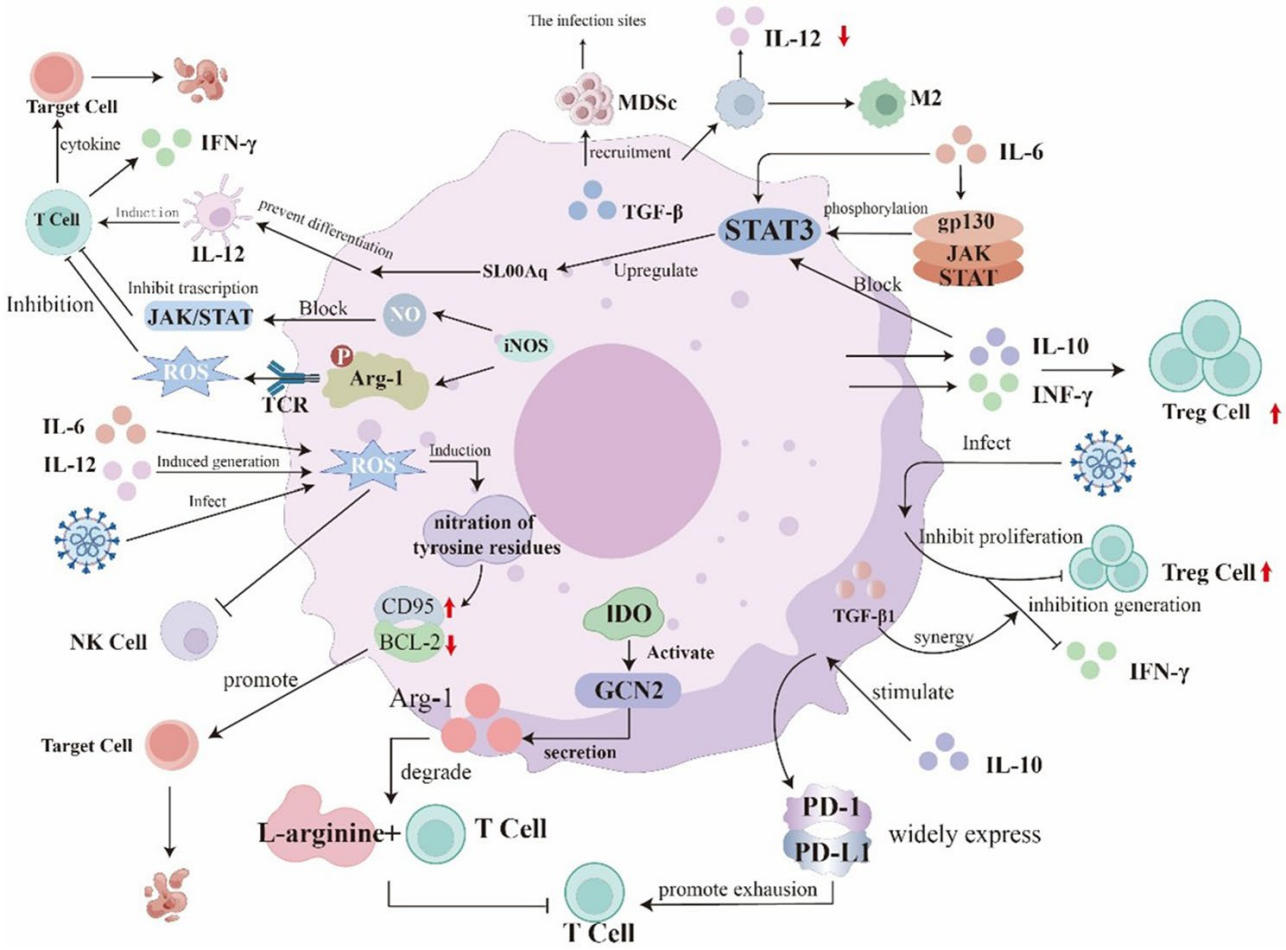
Mechanism of viral infection in MDSCs

MDSCs represent a diverse group of immature cells with a potent capacity to suppress T cell activity and regulate various innate and adaptive immune cells, including NK cells, macrophages, and dendritic cells [16, 107]. The proliferation of MDSCs correlates with the severity of lung diseases, as their numbers increase in response to pathological conditions such as viral respiratory infections and inflammation [15, 16]. These cells primarily originate from pre-hematopoietic somatic cells in the bone marrow and serve as progenitors for macrophages, dendritic cells, and granulocytes. In the context of viral infections, the normal differentiation of immature marrow cells is disrupted, leading to a significant rise in MDSC counts [15, 16]. MDSCs can be categorized into two main subgroups based on their surface markers, morphology, and functions: granulocyte or polymorphonuclear MDSCs (PMN-MDSCs or G-MDSCs), which resemble neutrophils, and monocyte MDSCs (M-MDSCs), which share characteristics with monocytes. Both subtypes possess the ability to inhibit T cell function [15, 108, 109]. While MDSCs are highly effective at suppressing T cells, they may exhibit a dual role during viral infections. On one hand, they can be detrimental to the host by inducing immunosuppression; on the other hand, they can contribute positively by maintaining immune homeostasis [17, 110]. Consequently, it is essential to approach the study of MDSCs in vivo with a balanced perspective [15] (Fig. 6, Supplementary Table 6).

#### Inflammation inhibition

Upon IAV infection, cytokines such as IL-6 and IL-10 play a crucial role in activating and recruiting MDSCs. IL-6 enhances the expression of the myeloid cell-associated protein S100A9 via the STAT3 signaling pathway, which inhibits the maturation of MDSCs into DCs, leading to the accumulation of MDSCs [18]. Furthermore, IL-6 inhibits the host immune response by phosphorylating STAT3 through the gp130/JAK/STAT pathway, which not only prevents the differentiation of MDSCs but also promotes their proliferation [16, 108]. In contrast, IL-10 mitigates the expansion of MDSCs by blocking IL-6 signal transduction via the STAT3 pathway. MDSCs activated by influenza A virus infections hinder T cell proliferation and IFN-γ production. Additionally, the IL-10 and TNF-α produced by these MDSCs facilitate the proliferation of regulatory T cells (Tregs), which diminishes the overall immune response in the body. Cytokines such as IL-6 and IL-10 also stimulate MDSCs to produce ROS. In activated cells, ROS can induce apoptosis in target cells by causing post-translational modifications of proteins (nitration of tyrosine residues) or by increasing CD95 expression while downregulating B-cell lymphoma-2(BCL-2) [18]. The proliferating MDSCs also produce TGF-β1, which contributes to their accumulation at infection sites. Additionally, TNF-α and IL-1β secreted by MDSCs can enhance the survival and persistence of these infected cells at the sites of infection [108].

#### Immunosuppressive effects

The increased proliferation of MDSCs during viral infection and are associated with disease severity. They accumulate more in critically ill or ICU COVID-19 patients. They participate in the pathogenesis of SARS-CoV-2 infection by promoting the secretion of anti-inflammatory cytokines, inhibiting the production of IFN-γ mediated by inducible nitric oxide synthase (iNOS), and suppressing T cell proliferation and response through mechanisms such as arginine deficiency, which ultimately delays viral clearance. The MDSCs-mediated immunosuppression in this viral infection occurs through multiple mechanisms, including: (1) IDO secreted by MDSCs can stimulate the reaction kinase General control nonderepressible 2(GCN2), while the secreted arginase 1 (Arg-1) degrades L-arginine and combines with T cell receptors (TCR) signals to co-inhibit the proliferation of T cells and promote the generation of Tregs; (2) MDSC-derived ROS and its downstream product, peroxynitrate, act on TCR, causing T cells to lose the ability to bind to phosphorylation MHCs and respond to specific antigens, leading to antigen-specific T cell tolerance. NO produced by iNOS suppresses IL-2 signaling in T cells by blocking the JAK–STAT pathway and induces apoptosis via cytokine nitration. In concert, iNOS and Arg-1 promote TCR nitrosylation and the formation of reactive nitrogen species, thereby impairing T cell function and survival; (3) Membrane-bound TGF-β1 produced by MDSCs inhibits the production of IFN-γ, and the produced IL-10 stimulates MDSCs to widely express programmed death protein 1 (PD-1) and programmed death ligand 1 (PD-L1), both of which jointly promote the occurrence and development of T cell exhaustion [15, 107].

#### Coordinated play

Bone marrow-derived MDSCs have the capacity to differentiate into mature dendritic cells, macrophages, or granulocytes. Under healthy conditions, MDSCs are typically present in minimal quantities. However, during pathological conditions, such as viral infections, their differentiation into mature myeloid cells is inhibited. MDSCs engage with various immune cells through multiple mechanisms to modulate their functions. These mechanisms include: (1) downregulating IL-12 production in macrophages and driving macrophage polarization into inhibitory M2 macrophages [15]; (2) In the case of viral infection, Instinctive NKT (iNKT) cells are deficient while MDSCs are increased. Adoptive transfer of iNKT cells can remove the inhibitory activity of MDSCs in vivo. Therefore, during viral infection, the interaction between iNKT cells and MDSCs regulates the inhibitory activity of MDSCs [110]; (3) viral-infected G-MDSCs inhibit NK cells by using ROS [18], impairing NK cell development, function, and cytotoxicity; (4) The development and functional activity of dendritic cells are impaired, compromising their intermediary role between innate and adaptive immune responses and reducing their ability to stimulate IFN-γ secretion in T cells [15]. In innate immunity and adaptive immune responses to viral infection, the inhibitory effect of MDSCs is mediated by multiple mechanisms besides the above reasons, including: (1) disruption of T cell homing via recombinant A disintegrin and metalloprotease 17(ADAM17) expression, and downregulation of l-selectin on naive T cells and e-selectin on the vasculature; (2) Attracting Tregs to an inflammation state by producing CCL4 and CCL5 chemokines; (3) Inducing and recruiting Th17 cells [15].

#### Adaptive immune cells

The adaptive immune system is primarily composed of cellular and humoral immunity, which involves antigen-specific antibodies generated by CD4 Th cells, CD8 cytotoxic T cells and B cells [43]. T cells play a crucial role in cellular immunity and exert significant antiviral effects [111]. They can be categorized into two main types: CD4^+^T cells and CD8^+^T cells. In the context of viral infections, the quantity of T cells and their subsets correlates with disease severity and prognosis [112]. B cells are pivotal in orchestrating humoral immune responses by producing antibodies that specifically neutralize pathogens [113]. Upon viral invasion, B cells generate virus-specific antibodies, while the efficiency of viral clearance hinges on the activity of specific CD4^+^ and CD8^+^T cells [111]. Both cell types possess cytotoxic activity, and it is through these cytotoxic T cells that effective immune responses against viral infections are mediated, allowing them to eliminate infected cells [114]. The intricate synergy and interaction among various components of adaptive immunity enhance the ability to control viral infections and enhance overall immunity.

### T cells

#### T cells and their functions

**Fig. 7:**
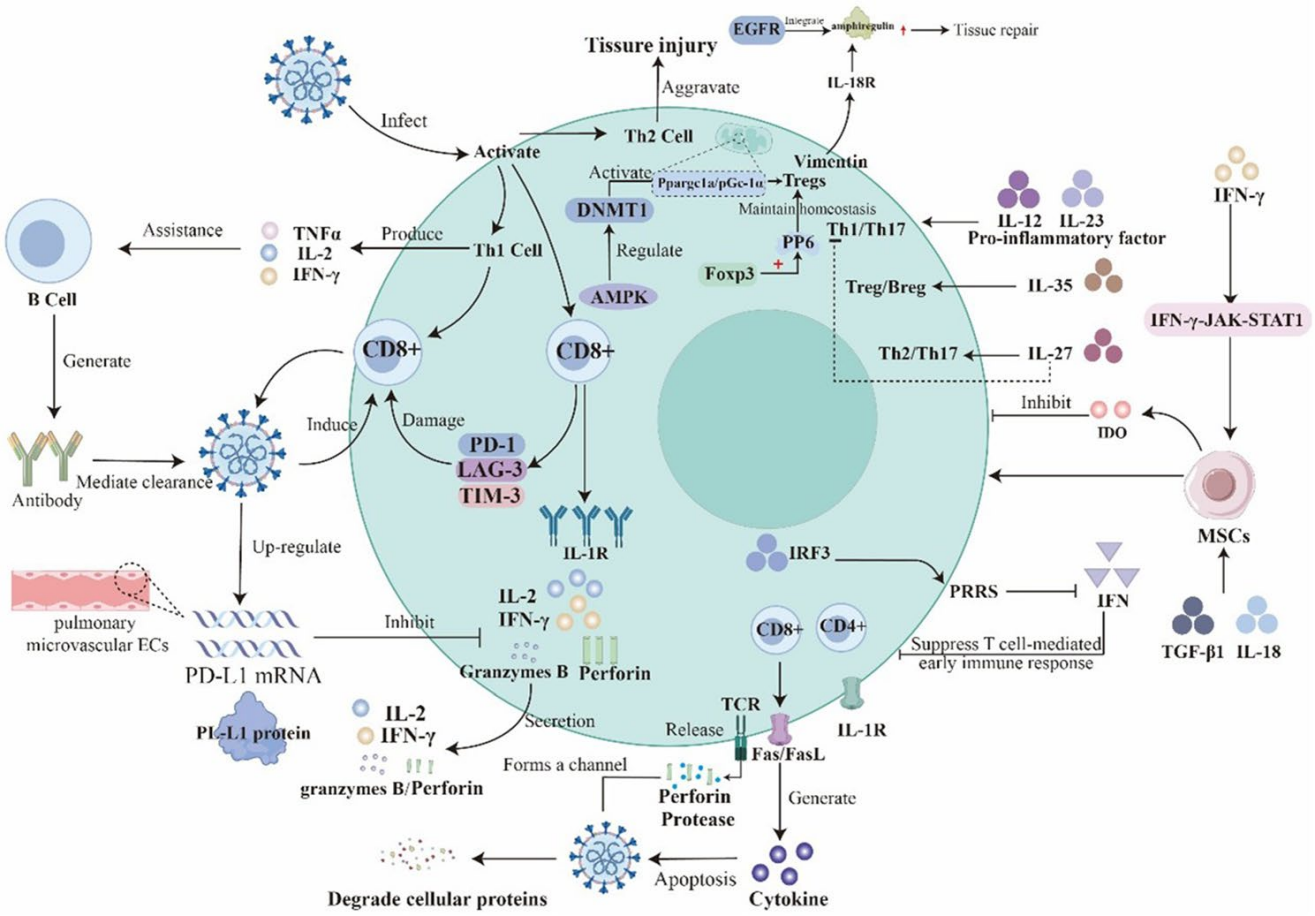
Mechanism of viral infection in T cells

T cell immunity is vital for the adaptive immune response to viral infections, primarily relying on the activation of pathogen-specific CD3^+^T cells, including CD8^+^T cells or cytotoxic T cells responsible for recognizing and destroying infected target cells, and CD4^+^T cells, which coordinate immune responses and facilitate B cell activation for antibody production [113]. During the acute phases of diseases such as SARS and MERS, there is a notable decrease in the counts of CD3^+^ and CD4^+^T cells. The number of CD3^+^T cells in severe patients is lower relative to that in mild patients [115]. In severe and critical COVID-19 patients, the number of peripheral CD4^+^ and CD8^+^T cells decreased significantly [116]. The extent of T cell depletion is closely associated with the severity of the disease [14, 115]. Therefore, the dynamic fluctuations in CD3^+^, CD4^+^, and CD8^+^T cell populations serve as crucial pathological markers for assessing viral infection and its severity (Fig. 7, Supplementary Table 7).

CD4^+^T cells enhance the functionality of CD8 T cells and B cells by releasing cytokines, while CD8^+^T cells play a crucial role in eliminating viruses by destroying infected cells [116, 117] Unlike antibodies, which mainly prevent viruses from entering cells, CD8 T cells directly target and kill virus-infected cells (e.g., those infected with influenza, CMV, or EBV) by recognizing viral peptides presented on these cells [118]. When influenza A virus invade the body, APCs display viral antigens on MHC I and MHC II molecules [119]. Endogenously processed viral peptides are presented by MHC I molecules on the surfaces of infected cells, where they are recognized by initial CD8 T cells, prompting their differentiation into cytotoxic T cells capable of killing infected cells through cytokine secretion and the release of cytotoxic granulates. Conversely, exogenously processed viral peptides are presented by MHC II on the surfaces of infected cells and are recognized by initial CD4 T cells. This recognition initiates distinct differentiation pathways, that lead to the formation of various effector subsets with specialized immune functions, such as Th1 cells, Th2 cells, aTh17 cells and Treg cells. Viral infection activates Th1 cells, producing cytokines such as TNF-α, IL-2 and IFN-γ. IFN-γ plays an antiviral role through direct cytotoxicity mechanisms and can mediate viral clearance by assisting B cells in producing antibodies. Th1 cells that secrete IFN-γ are essential for activating CD8 T cells and facilitating viral clearance. In contrast, Th2 cell-mediated immune responses can exacerbate lung tissue damage and delay viral clearance. Additionally, Th17 cells, which are induced by viral antigens, contribute to the virus’s pathogenicity and worsen lung inflammation following a viral attack. However, the virus also triggers Tregs to migrate to the lungs, resulting in a reduction of Th17 cell numbers and a subsequent alleviation of pulmonary inflammation [43, 87, 120].

#### Regulatory T cells

Tregs, a subtype of CD4^+^T cells, play a vital role in promoting self-tolerance and maintaining immune homeostasis. They facilitate tissue recovery and repair following pulmonary injury caused by IAV infection through the production of amphiregulin, an epidermal growth factor receptor (EGFR) ligand. However, the deletion of Tregs can hinder viral clearance in the presence of increased T cell recruitment and IFN-γ [119]. Vimentin, a significant protein within the distal polar complex (DPC) of Tregs, plays a crucial role in this process. It inhibits the induction of amphiregulin by sequestering the interleukin-18 receptor (IL-18R) within the DPC, which limits the necessary receptor-ligand interactions for tissue repair. Consequently, in cases of severe viral pneumonia, Tregs lacking vimentin contribute to reduced pulmonary injury and mortality associated with IAV infection. The conditional knockout of vimentin alters the spatial distribution of Treg surface proteins, enhances the inhibitory functions of CD39 and CD73, and accelerates the relief of pulmonary inflammation via the CD73-adenosine signaling pathway [121]. Severe SARS-CoV-2 viral infection can lead to T cell dysregulation, and excessive release of T cell-mediated cytokines can lead to systemic inflammation and tissue damage. Under inflammation conditions, Treg cells may downregulate FoxP3 expression and adopt a pathogenic phenotype associated with proinflammatory cytokines. The pp6 protein, a key positive regulator of FoxP3, is essential for maintaining Treg cell stability and preventing immune dysregulation [116]. The functionality of Treg cells is also closely tied to mitochondrial metabolism. AMPK, is an epigenetic regulatory factor that supports metabolic functions and transcriptional programs of the Treg lineage. During IV viral pneumonia, AMPK modulates DNA methyltransferase 1(DNMT1) to activate the expression of the metabolic gene Ppargc1a/PGC-1α, which is critical for mitochondrial function. When AMPK is deleted from Treg cells, it leads to hypermethylation of key metabolic gene promoters. Therefore, AMPK plays a pivotal role in determining the immune response of Treg cells, especially in a metabolically stressful microenvironment [122].

#### T cell reduction and exhaustion

RSV or SARS-CoV-2 infections can induce T cell exhaustion, particularly evident during acute viral infections. Lung CD8^+^T cells exhibit upregulation of several inhibitory receptors, including PD-1, lymphocyte activation gene 3 (LAG-3), and T cell immunoglobulin domain and mucin domain-3 (TIM-3). These inhibitory receptors contribute to the impairment of CD8^+^T cells during viral infections. Interestingly, when PD-1 is deleted, blocking LAG-3 can restore the effector functions of CD8^+^T cells but may exacerbate lung pathology, highlighting the LAG-3’s protective role against immune pathology during viral clearance [14, 123]. Elevated levels of PD-1 and TIM-3 serve as indicators of T cell exhaustion and correlate with the severity of viral infections [14]. PD-L1 is expressed on activated T cells, B cells, macrophages and vascular endothelial cells (ECs). RSV or H9N2 virus infection upregulates the expression of PD-L1 mRNA and PD-L1 protein levels in pulmonary microvascular ECs, inhibits the immune response of T cells, and significantly reduces the secretion of IL-2, IFN-γ, and granzyme B, along with perforin expression in T cells, thereby promoting viral immune escape [124].

#### Mechanism of action of T cells

(1) Regulatory effects of interleukins on T cells. The expression of IL-1 receptor complex on CD4 T cell subsets has been widely reported. Indeed, CD8 T cells dynamically upregulate their responsiveness to IL-1 during APC priming, with memory CD8 T cells exhibiting elevated IL-1R1 levels. Consequently, the lack of IL-1 signal transduction during respiratory viral infections limits the expansion of anti-viral CD8 T cell immunity [125]. IL-3 is a predictive marker of infection severity and outcomes, enhancing immune function during viral pneumonia by promoting pDC-mediated T cell activation [95]. The IL-12 family, which includes IL-12, IL-23, IL-27, IL-35 and IL-39, plays a crucial role in the induction and regulation of both innate and adaptive immune responses. IL-12 and IL-23 are proinflammatory and contribute to the differentiation of helper T cell subsets Th1 and Th17, respectively. IL-35 is produced by Treg and Breg cells. IL-27 induces the development of Th1 cells and inhibits the differentiation of Th2 and Th17 cells. IL-27 signaling regulates T cell-mediated immunity by enhancing virus-specific CD4 T cells [126].

(2) Regulatory effects of mesenchymal stem cells on T cells. MSCs are non-hematopoietic cells with immunomodulatory capabilities that can suppress inflammation triggered by influenza viruses. In their resting state, MSCs do not express IDO, but upon activation by cytokines (IFN-γ, TNF-α, IL-1β, IL-17 A and IL-25) in inflammatory microenvironments, they overexpress IDO, exerting immunosuppressive effect. For example, IFN-γ induces IDO expression in MSCs via the IFN-γ-JAK-STAT1 pathway, inhibiting T cell proliferation and inducing apoptosis. IL-17 A enhances the proportion of Treg cells through the COX-2/PGE2 pathway, while IL-18 stimulates MSCs via a TGF-β1-mediated pathway to amplify T cell suppression both in vitro and in vivo. MSCs overexpressing TGF-β1 can modulate pulmonary inflammation and alleviate lung injury by correcting the Th17/Treg imbalance within lung tissue [127].

(2) T cell-mediated cell apoptosis. During viral infections, CD8^+^ cytotoxic T cells are activated through TCR and additional co-stimulatory signals, releasing perforin and proteases to form channels in the membranes of target cells, leading to the degradation of cellular proteins and the elimination of infected cells. Additionally, CD8^+^ cytotoxic T cells can trigger apoptosis in target cells through the Fas/FasL pathway. The release of immunomodulatory cytokines (IFN-γ, TNF and IL-10) also directly participate in anti-virus responses [119, 128, 129]. Activation of the NLRP3 inflammasome can further activate T cell apoptosis. Therefore, targeting the downstream mediators of NLRP3 inflammasome activation, such as caspase-1 and cytokines IL-1β and IL-18, represents a potential therapeutic approach for treating viral pneumonia [130].

(4) The F protein is a primary pathogenic factor of RSV. F protein interacts with nucleolin (NCL), epidermal growth factor receptor (EGFR), insulin-like growth factor 1 receptor (IGF1R) and intercellular adhesion molecules 1 (ICAM1), enabling the fusion of host and viral plasma membranes, ensuring the entry of viral RNA into host cells. During the fusion process, F protein transitions from a pre-fusion (preF) to a post-fusion conformation (postF), which activates a cytotoxic T cell response [131]. Proteomic analyses of viral-infected lower respiratory tracts have shown that viruses inhibit the production and function of PRRs by inhibiting the activation and translocation of IRF-3, thus blocking interferon synthesis and secretion, which suppresses the induction of early adaptive immune responses by T cells [132].

#### B cells

**Fig. 8:**
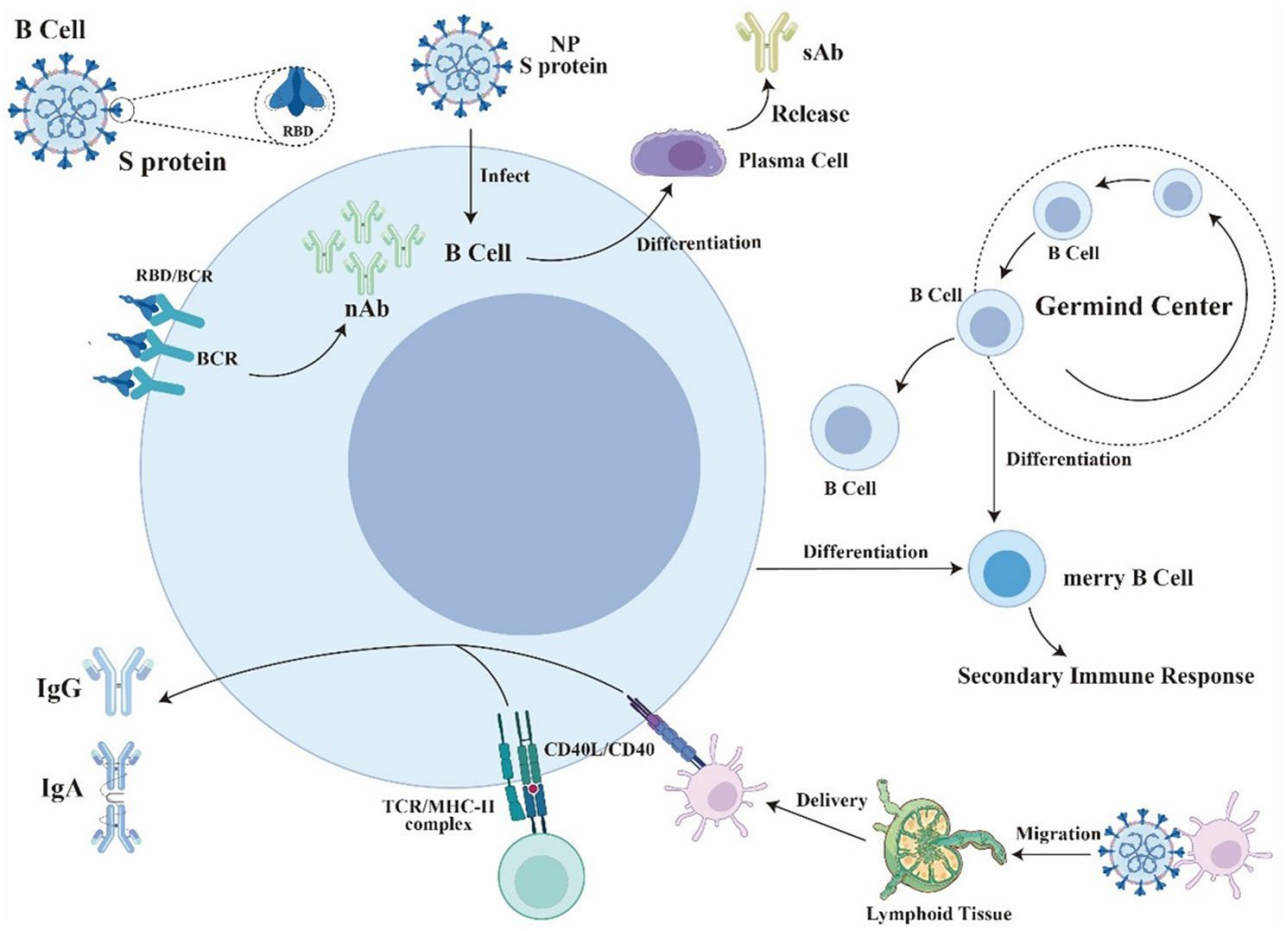
Mechanism of viral infection in B cells

B cells are essential components of adaptive immune responses, responsible for antibody production, antigen presentation, and cytokine secretion to regulate immune responses [133, 134], which is a crucial role in the human immune system’s defense against viral infections. Following respiratory viral infections, B cells and the antibodies they produce significantly influence the duration and effectiveness of the immune response [135, 136] (Fig. 8).

#### B cells and their functions

B cells originate from bone marrow stem cells [133]. Upon encountering viral antigens, primarily from spike proteins and nucleocapsid proteins, B cells differentiate into plasma cells, which produce antibodies specific to these viral antigens [135]. Neutralizing antibodies, a hallmark of immune protection against viral pneumonia, are critical effector molecules generated by B cells in response to viral infections [137]. Respiratory viral infections can elicit robust B cell responses, leading to the production of substantial amounts of virus-specific IgM, IgA and neutralizing IgG antibodies [138]. These antibodies not only prevent viral entry into host cells but also facilitate the clearance of infected cells through opsonization and subsequent phagocytosis [139]. Although viral infections prompt B cells to produce specific antibodies, this response wanes once the pathogens are controlled, resulting in the majority of pathogen-specific B cells undergoing apoptosis [140]. Nevertheless, during the initial infection phase, B cells mature within germinal centers (GC), generating long-lived plasma cells that secrete neutralizing antibodies. Additionally, B cells can differentiate into memory B cells, which are rapidly reactivated upon subsequent infections. These memory B cells are vital for establishing long-term protective immunity against viral pneumonia [141, 142].

#### Regulatory effects on B cells during viral infection

(1) Targets and mechanisms of action of neutralizing antibodies. During SARS-CoV-2 infections, B cells rapidly generate neutralizing antibodies that target spike proteins on the virus surface, with the RBD being the primary target. Neutralizing epitopes on the SARS-CoV-2 RBD, particularly those corresponding to the ACE2 receptor binding footprint, are highly immunogenic and readily recognized by antibodies. Consequently, antibodies bind to viral surface proteins directly neutralizing the virus and preventing its entry into host cells [135].

(2) Assisting the removal of viruses. During viral infections, mature DCs migrate to secondary and tertiary lymphoid tissues, carrying viral antigens that they present to B cells traversing these tissues. CD4^+^ helper T cells recognize B cells via the cognate antigens presented by MHC class II molecules and promote class switching to IgG and IgA by interacting with the costimulatory receptor CD40 and its CCD40L receptor, as well as signal transduction via cytokines (e.g., IL-4, IFN-γ, TGF-β). Specific CD4^+^ TFH cells expressing the costimulatory receptor ICOS and molecules like PD-1 and IL-21, as well as cells driven by the transcription repressor Bcl-6, promote somatic hypermutation and affinity maturation. Therefore, a large number of high-affinity antibodies to neutralize viruses. After antigen clearance, some plasma cells differentiate into long-lived plasma cells that migrate to the bone marrow and respiratory tract, where they maintain immunoglobulin production through TLR signalling (TLR9 and TLR10) on B cells to sustain long-term antibody levels in vivo. Upon re-infection with the same antigen, memory B cells undergo secondary activation, leading to rapid proliferation and antibody production. Additionally, some cells re-enter the GC center to further enhance affinity maturation and replenish the memory pool, enabling responses to recurrent respiratory viral infections [143].

## Clinical and translational perspectives

Immunocellular dysregulation plays a dual role in the progression of viral pneumonia: a moderate immune response aids in viral clearance and tissue repair, while dysfunctions such as cytokine storms [144], excessive release of NETs [12], hyperproliferation of MDSCs [15], and T-cell exhaustion [14] can lead to immunopathology and tissue damage [145]. These dysfunctions significantly contribute to the exacerbationof the disease. Consequently, restoring immune homeostasis is crucial for developing personalized immunotherapy strategies.

Current therapeutic approaches to immune dysregulation are increasingly focused on the precise modulation of key immune cells. For instance, JAK inhibitors like baricitinib inhibit excessive macrophage activation through the JAK-STAT signaling pathway, effectively suppressing cytokine storms and receiving emergency use authorization for moderate-to-severe COVID-19 patients [144]. IL-6 receptor antagonists (e.g., tocilizumab) significantly lower mortality rates in severe cases by blocking the IL-6 signaling pathway [145]. Furthermore, PD-1/PD-L1 immune checkpoint inhibitors help reverse T-cell exhaustion and restore T-cell function, maintaining immune responses by inhibiting the overactivation of the PD-1/PD-L1 pathway during viral infections [124]. The abnormal expansion of MDSCs is a central mechanism contributing to persistent immunosuppressive disorders [146]. Studies have revealed that MDSCs inhibit T cell proliferation and response through various mechanisms, including the secretion of Arg-1, iNOS, and ROS, as well as inducing anti-inflammatory factor secretion, all of which delay viral clearance [15, 107]. Consequently, targeted therapies targeting MDSCs—such as Arg-1 inhibitors to block their immunosuppressive functions or strategies to promote their differentiation into mature myeloid cells—are currently undergoing clinical trials for tumor immunotherapyand show promise for application in viral pneumonia [15]. Recent findings have underscored the importance of cell death pathways—including apoptosis, pyroptosis, necroptosis, and ferroptosis—in the immunopathology of viral pneumonia [147]. The excessive release of pathogen-associated molecular patterns (PAMPs) and damage-associated molecular patterns (DAMPs) disrupts both positive and negative feedback loops, amplifying inflammatory responses [144]. Accordingly, clinical studies indicate that caspase inhibitors and RIPK1 inhibitors may help alleviate lung injury by targeting these mechanisms [147].

Achieving precise interventions for immune dysregulation necessitates the establishment of a reliable biomarker system that allows for the dynamic assessment of patients’ immune status. Currently validated clinical indicators include: lymphocyte counts and their subsets [14], the neutrophil/lymphocyte ratio (NLR) [148, 149], levels of inflammatory cytokines, and C-reactive protein (CRP) [145]. Additionally, advancements in single-cell sequencing technology have uncovered the heterogeneity and functional states of myeloid cells, offering new tools for precise immune profiling [150]. Integrating multi-omics data with clinical information to create personalized immune status assessment models represents a critical pathway for translating mechanistic insights into therapeutic interventions.

Despite significant advancements in understanding the mechanisms underlying immune cell dysregulation, several gaps persist. The dysregulation induced by viral pneumonia is a complex and evolving process involving multiple immune cells, with unclear dynamic interactions at different stages of infection, and myriad of knowledge gaps concerning individual variability [145]. Future research must prioritize the integration of multi-timepoint sampling and single-cell multi-omics technologies to create precise and comprehensive dynamic profiles of immune dysregulation. The same immune phenotype (e.g., MDSC expansion) might originate from distinct molecular mechanisms and display markedly different therapeutic responses [15].Therefore, it is vital to develop a deep understanding of the molecular subtypes of immune cell dysregulation and to establish a specific mechanistic classification system. Monotherapy targeting a single mechanism often fails to reverse complex states of immune dysregulation effectively. Future investigations should evaluate the combination or optimization of immunomodulators, antiviral agents, and supportive therapies to achieve synergistic effects [144]. Recent studies have also shown that innate immune cells can develop long-term enhanced antiviral capabilities through “trained immunity.” Inducing trained immunity via vaccines or immunomodulators may present a novel strategy to strengthen antiviral defenses and prevent severe outcomes [151, 152].

## Conclusions

The immunological disorder caused by viral pneumonia is a complex and dynamically evolving phenomenon involving multiple immune cells. The coordinated activity of these immune cells aims to clear viruses and establish immunological memory. However, if the response is too strong, out of control or uncoordinated, such as cytokine storm, excessive release of NETs, excessive suppression of MDSCs, T cell exhaustion, etc., it will lead to immune pathological damage and become an important cause of disease aggravation. This study systematically summarizes the mechanistic roles of immune cells in viral pneumonia and explores current therapeutic strategies for immune dysregulation from clinical and translational perspectives, highlighting the progressive shift toward precise regulation of key immune cells. However, significant gaps remain, including unclear dynamic interaction mechanisms of immune cells across infection stages, individual variability, and the lack of composite or optimized combination therapies. Future research urgently requires single-cell multi-omics approaches and dynamic immune monitoring to gain deeper insights into immune regulatory networks. Therefore, future studies should exploit the regulatory mechanisms of these immune cells to restore immune homeostasis, rather than merely suppressing or enhancing immune responses, marking a significant direction for precision immune interventions in respiratory viral infections.

## Electronic Supplementary Material

Below is the link to the electronic supplementary material.

Supplementary Material 1

## Data Availability

No datasets were generated or analysed during the current study.

## Abbreviations

CAP: Community-acquired pneumonia
SARS: Severe acute respiratory syndrome
MERS: Middle east respiratory syndrome
COVID-19: Corona virus disease-2019
DC: Dendritic
NK: Natural killer
PRRs: Pattern recognition receptors
TNF-α: Tumor necrosis factor-α
IFN-γ: Interferon-γ
MHC: Major histocompatibility complex
TRAIL: TNF-related apoptosis-inducing ligand
FasL: Factor related apoptosis ligand
NKG2D: Natural killer group 2
IL-6/10/21: Interleukin-6/10/21
TGF-β: Transforming growth factor
APCs: Antigen-presenting cells
KIRs: Killer-cell immunoglobulin-like receptors
CXCR3: C-X-C motif receptor 3
CCR5: Chemokine (C-C motif) receptor 5
CXCL9/10/11: Chemokine (C-X-C motif) ligand 9/10/11
STAT3/4: Signal transducer and activator of transcription 3/4
CCL3/4: C-C motif chemokine ligand 3/4
MSCs: Mesenchymal stem cells
IDO: Indoleamine 2, 3-dioxygenase
PGE2: Prostaglandin E2
PAMP: Pathogen associated molecular patterns
TLR4: Toll-like receptor 4
NF-κB: Nuclear factor kappa B
IRF3: Interferon regulatory factor 3
NLRP3: NOD-like receptor pyrin domain containing 3
ACE2: Angiotensin-converting enzyme 2
DAMPs: Damage-associated molecular patterns
IAV: Influenza virus A
HIF-1α: Hypoxia-inducible factor-1α
AMPK: Adenosine monophosphate-activated protein kinase
Plet1: Placenta-expressed transcript 1
MAPK: Mitogen-activated protein kinase
TAK1: Transforming growth factor-beta-activated kinase 1
GABA: Gamma-aminobutyric acid
TFAM: Transcription factor A
GM-CSF: Granulocyte-macrophage colony-stimulating factor
PPAR-γ: Peroxisome proliferator activated receptor gamma
RSV: Respiratory syncytial virus
ROS: Reactive oxygen species
NETs: Neutrophil extracellular traps
PMN: Polymorphonuclear neutrophil
NSPs: Neutrophil serine proteases
ARDS: Acute respiratory distress syndrome
ALI: Acute lung injury
NADPH: Nicotinamide adenine dinucleotide phosphate
LDHB: Lactate dehydrogenase B
MDSCs: Myeloid-derived suppressor cells
iNOS: Inducible nitric oxide synthase
GCN2: General control nonderepressible 2
PD-1: Programmed death protein 1
PD-L1: Programmed death ligand 1
DNMT1: DNA methyltransferase 1
LAG-3: Lymphocyte activation gene 3
TIM-3: T cell immunoglobulin domain and mucin domain-3
EGFR: Epidermal growth factor receptor
IGF1R: Insulin-like growth factor 1 receptor
ICAM1: Intercellular adhesion molecules 1
