# Supplementary material for: Immune cell disorder in viral pneumonia
Source: J Transl Med. 2026 May 16;24:884. doi: 10.1186/s12967-026-08193-z (PMC13360541; doi:10.1186/s12967-026-08193-z)
Supplement: Supplementary file 1 — Supplementary Material 1 [file 12967_2026_8193_MOESM1_ESM.doc]

**Supplementary Table 1** Correspondence between immune dysfunction and immune cells, pathological mechanisms

| Type | Core immune cells | Key Pathological Mechanisms | Representative molecule/marker | Pathological consequences |
| --- | --- | --- | --- | --- |
| Hyperactivated type | Macrophages, neutrophils, NK cells | Regulation of cytokine storm, excessive release of NETs | IL-6,TNF-α,IFN-γ,NETs | Pulmonary injury, multiple organ failure |
| Inhibition of persistence | T cells, MDSCs, NK cells | T cell depletion, MDSC expansion | PD-1,TIM-3,Arg-1,ROS and IL-10 | Persistent viral presence and secondary infection |
| Functional defect type | DCs, macrophages, NK cells, T cells, and B cells | Antigen presentation dysfunction, initial immune response failure | IFN-α/β,antibody | Early uncontrolled infection progression to severe disease |

**Supplementary Table 2** Comparison of NK cells with different viral infections

| Virus type | NK cell function | Interaction process | NK cell status | Active ingredient | Result | References |
| --- | --- | --- | --- | --- | --- | --- |
| SARS-COV-2 | Natural cytotoxic receptors (NKp30, NKp44, and NKp46), type C lectin-like receptors (NKG2D and NKp80), and co-activating receptors (DNAM1 and CD2) | Modulated by cytokines (IL-2/12/15/18, IFN-1) | NK cells are activated | Through Ca2+-dependent lytic granules (granulase/perforin), or via death receptors (such as TRAIL and FasL) | Mediate target cell apoptosis | 31 |
|  | Natural cytotoxic receptors (NKp30, NKp44, and NKp46), type C lectin-like receptors (NKG2D and NKp80), and co-activating receptors (DNAM1 and CD2) | Increased cytokines levels(IL-6, IL-10, and TGF-β) associated with severe COVID-19 | Inhibition of NK cells | Suppresses the synthesis of IFN-γ and granulase B | NK cell depletion | 28 |
|  | High expression of STAT3 | Promotes the production of IL-21 by activated CD4+ T cells | Stimulates | Stimulates downstream activation of c-Myc | Promotes NK cell proliferation | 36 |
|  | PD-1,TIM-3 | Virus triggers the transformation of NK cells into exhausted phenotype NK cells | Activation and depletion | IL-6 | Suppressing host immunity | 14 |

**Supplementary Table 2** (continued)

| Virus type | NK cell function | Interaction process | NK cell status | Active ingredient | Result | References |
| --- | --- | --- | --- | --- | --- | --- |
|  | TLR3 receptor | IFN-γ-activated mesenchymal stem cells enhance the expression of HLA-I-like molecules, thereby interacting with the killer cell immunoglobulin-like receptor (KIR). In this manner, NK cell-mediated cytolytic activity is inhibited. | Inhibit | TGF-β1 and HGF molecules | Immunosuppression of NK cells | 38 |
|  |  | Viral infection induces phenotypic and functional alterations as well as differentiation of NK cells (e.g., CD56DimCD57+) and promotes their homing to affected organs. | Activation and amplification | IL-12, IL-18, and Interferon type I | Antivirus | 33 |
| vaccinia virus (VACV) | Transcription and modification of IFN-γ genes during early bone marrow development | During VACV infection | Presents as a highly activated phenotype | Generate the cytokine IFN-γ | Suppressing the replication of VACV and its transmission from the lungs | 29 |
| RSV | High expression of activated receptors NKG2D and CD27 | In the early stage of RSV infection | Activation and aggregation | Synthesize the cytokine IFN-γ | Exacerbates immune damage to pulmonary tissues | 30 |

**Supplementary Table 2** (continued)

| Virus type | NK cell function | Interaction process | NK cell status | Active ingredient | Result | References |
| --- | --- | --- | --- | --- | --- | --- |
| cytomegalovirus (CMV) | surface receptor | Recognition of infected cells through stress ligands or pathogen-associated molecular patterns | NK cells are activated | Release of granulase/perforin, or via death receptors | Mediate target cell apoptosis | 28 |
|  | High expression of CD96 | Mediates protein tyrosine kinase Zap70 and/or Syk phosphorylation | Activate | Activation of Ly49H (an NK receptor specific to MCMV m157 protein) enables specific recognition of the m157 protein on the viral surface. | Stimulates the antiviral activity of NK cells | 32 |
| IAV | CXCR3, CCR5 chemokine receptors | The natural cytotoxic receptor of NK cells binds to influenza virus hemagglutinin | The function of NK cells is activated | Through CD6-mediated antibody-dependent cell-mediated cytotoxicity (ADCC) | Clear infected cells | 34 |
| IV | Expressing CXCR3, CXCR6, and CCR5 chemokine receptors | Viral infection stimulates phenotypic and functional changes as well as differentiation of NK cells (e.g., CD56DimCD57+) and stimulates their homing to affected organs. | Activation and amplification | IL-12, IL-18, and Interferon type I | Antivirus | 33 |

**Supplementary Table 2** (continued)

| Virus type | NK cell function | Interaction process | NK cell status | Active ingredient | Result | References |
| --- | --- | --- | --- | --- | --- | --- |
| Human cytomegalovirus (CMV) | Upregulation of CD69 | Virus activation of pDCs induces upregulation of the NK cell activation marker CD96 | Activate | Secretion of soluble Fas ligand, perforin, and IFN-γ | Inhibits viruses | 91 |

**Supplementary Table 3** Comparison of Macrophages with Different Viral Infections

| Virus type | Macrophage function | Interaction process | Macrophage status | Active ingredient | Result | References |
| --- | --- | --- | --- | --- | --- | --- |
| SARS-COV-2 | ACE2 receptor | The SARS-CoV-2 S protein binds to the ACE2 receptor, stimulating excessive TLR signaling response in macrophages and suppressing IRAK-M. | Activate | Promotes overexpression of pro-inflammatory factors such as IL-6 and TNF-α | Cytokine Storm and Pulmonary Injury | 47 |
|  | RIG-I-like receptor | SARS-CoV-2 nucleoprotein interferes with RIG-I-mediated activation of the mitochondrial antiviral signaling protein MAVS via its helicase domain | Inhibit | Suppression of type I interferon production | Enhance viral replication | 46, 48 |
|  | Express T cell chemokines | SARS-CoV-2 infects alveolar macrophages, which express T cell chemokines. Activated T cells recognize SARS-CoV-2 antigens presented by alveolar macrophages. | Activate | The produced IFN-γ further activates alveolar macrophages to generate cytokines and chemokines; activated T cells proliferate and continuously produce IFN-γ, thereby causing death of infected TRAMs and the recruitment of monocytes. These monocytes differentiate into monocyte-derived alveolar macrophages (MoAM), which are recruited to infect SARS-CoV-2 and continue to present antigens to T cells. | Virus clearance | 50 |

**Supplementary Table 3** (continued)

| Virus type | Macrophage function | Interaction process | Macrophage status | Active ingredient | Result | References |
| --- | --- | --- | --- | --- | --- | --- |
|  | Highly active HIF-1α | Viral infection activates the Wnt-β-catenin-HIF-1α signaling pathway in macrophages | inflammatory | Promotes AM to generate pro-inflammatory mediators | Inhibit the proliferation and self-renewal of AMs;promote inflammatory AMs | 52 |
|  | Telomere shortening | Macrophages undergo senescence-like transformation, demonstrating mitochondrial distress and abnormal activation of the STING and NLRP3 inflammasome pathways. | Involution form |  | Severe condition | 58 |
|  | Express GABA receptors | GABA activates macrophages via its receptors |  | Expression of key molecules stimulating mtDNA replication and mitochondrial biogenesis (e.g., SIRT1, PGC-1α, and TFAM) | Restoring tissue homeostasis | 42 |
|  |  | Induction of Th1 cytokines | M1 type | Release of pro-inflammatory factors such as TNF-α, IL-1β, IL-12, and iNOS | Enhance inflammatory response | 54 |
|  |  | Promotes Th2 cytokines | M2 type | Release immune regulatory factors such as Arg-1, IL-10, and TGF-β | Reduce inflammatory response | 54 |

**Supplementary Table 3** (continued)

| Virus type | Macrophage function | Interaction process | Macrophage status | Active ingredient | Result | References |
| --- | --- | --- | --- | --- | --- | --- |
|  | STAT3 | The activation of signal transducer and transcription factor-3 (STAT3) occurs through cell-cell interactions between macrophages and monocyte-derived macrophages (MCSs). | M2 type | IL-10 | Promote tissue repair | 38 |
|  | TLR4 | In viral infections, the expression of secretory matrix cell protein Sparcl1 is elevated in pulmonary capillary endothelial cells, which acts on macrophages via TLR4. | M1 type | Expression of pro-inflammatory cytokines (such as TNF, IL-1β, IL-6) | Promotes inflammatory response | 13 |
|  | TAK1 | Suppression of NF-κB phosphorylation/activation | Inhibition of M1 type | Reduces the release of pro-inflammatory cytokines such as IL-1β, IL-6, and TNF-α | Inhibit inflammation and alleviate pulmonary injury | 55 |
| RSV | RIG-I-MAVS complex | During RSV infection, alveolar macrophages activate the RIG-I-MAVS complex | Activate | Generation of interferon type I, leading to enhanced recruitment of inflammatory monocytes | Restrict viral replication | 48 |

**Supplementary Table 3** (continued)

| Virus type | Macrophage function | Interaction process | Macrophage status | Active ingredient | Result | References |
| --- | --- | --- | --- | --- | --- | --- |
|  | TAK1 | Inhibition of NF-κB phosphorylation/activation | Inhibition of M1 type | Inhibits the release of pro-inflammatory cytokines such as IL-1β, IL-6, and TNF-α | Inhibit inflammation and alleviate pulmonary injury | 55 |
| IAV | High expression of CD206 |  | M2 type | Elevates Arg-1 with strong phagocytic capacity | Exerts protective effects in influenza virus infection | 43 |
|  | Expression of TLR3/4 receptors | Reveal the viral RNA structure and induce NF-κB and IRF3 |  | Induces IFN-I production, activates the JAK/STAT pathway, and induces the expression of ISGs | Inhibition of viral replication | 43 |
|  | ZBP1 | Recognition of viral riboproteins triggers the activation of NLRP3 inflammasome |  | Release of IL-1β and IL-18 | Inhibition of viral replication | 43 |

**Supplementary Table 3** (continued)

| Virus type | Macrophage function | Interaction process | Macrophage status | Active ingredient | Result | References |
| --- | --- | --- | --- | --- | --- | --- |
|  | Highly active HIF-1α | Viral infection activates the Wnt-β-catenin-HIF-1α signaling pathway in macrophages | inflammatory | Stimulates AM to produce pro-inflammatory mediators | Inhibit the proliferation and self-renewal of AMs;promote inflammatory AMs | 52 |
|  | High expression of placental transcript 1 (Plet1) gene | Stimulation of AEC proliferation via MEK and Src kinase signaling pathways |  | Synthesize Plet1 | Restoration of alveolar epithelial barrier function | 56 |
|  | Telomere shortening | Macrophages undergo senescence-like transformation, exhibiting mitochondrial distress and abnormal activation of the STING and NLRP3 inflammasome pathways. |  |  | Severe condition | 58 |
|  | Express GABA receptors | GABA activates macrophages via its receptors |  | Expression of key molecules enhancing mtDNA replication and mitochondrial biogenesis (e.g., SIRT1, PGC-1α, and TFAM) | Restoring tissue homeostasis | 42 |

**Supplementary Table 3** (continued)

| Virus type | Macrophage function | Interaction process | Macrophage status | Active ingredient | Result | References |
| --- | --- | --- | --- | --- | --- | --- |
| IV | Express IFN-β | IFN-β expressed by alveolar macrophages is secreted through autocrine mechanisms. |  | Apoptosis-inducing factor TNF-related apoptosis-inducing ligand (TRAIL) | Promote alveolar epithelial cell (AEC) injury | 49 |
|  | Low expression of Mint3 | Mint3-deficient macrophages inhibits HIF-1 and NF-κB signaling pathways |  | Modulate the production of inflammatory cytokines/chemokines in macrophages | Exert protective effects | 53 |
|  | TLR4 | In viral infections, the expression of secretory matrix cell protein Sparcl1 is upregulated in pulmonary capillary endothelial cells, which acts on macrophages via TLR4. | M1 type | Expression of pro-inflammatory cytokines (such as TNF, IL-1β, IL-6) | Promote inflammatory response | 13 |
|  | TAK1 | Suppression of NF-κB phosphorylation/activation | Inhibition of M1 type | Block the release of pro-inflammatory cytokines such as IL-1β, IL-6, and TNF-α | Inhibit inflammation and alleviate pulmonary injury | 55 |
| H1N1 | MAPK signal channel | IL-17 polarizes macrophages from M1 to M2 type via the MAPK pathway, particularly the ERK1/1 and p38 pathways. | M2 type | Inhibition of pro-inflammatory factors | Alleviate lung injury and promote tissue repair | 57 |

**Supplementary Table 3** (continued)

| Virus type | Macrophage function | Interaction process | Macrophage status | Active ingredient | Result | References |
| --- | --- | --- | --- | --- | --- | --- |
| EB virus | Secrete IL-1,TNF-α | Induce Th17 lymphocytes to produce IL-17A, which stimulates macrophages to secrete IL-1 and TNF-α. | Activate | IL-1,TNF-α | Reduction of tissue inflammation | 93 |

**Supplementary Table 4** Comparison of neutrophils with different viral infections

| Virus type | Neutrophil Function | Interaction process | Neutrophil status | Active ingredient | Result | References |
| --- | --- | --- | --- | --- | --- | --- |
| SARS-COV-2 | Release NETs | SARS-CoV-2 directly stimulates neutrophils to release NETs through the ACE2-serine protease activity axis, and activates TLR2/4/9 to exert cytotoxic effects. | Activate | Promotes the generation of cytokines such as IL-1β, TNF-α, and IL-6 | Promote inflammatory responses and lung injury | 12 |
|  | Release NETs | The antimicrobial peptides and myeloperoxidase of NETs induce chemotaxis of immune cells, promoting macrophage polarization toward M1. | Activate | Secretion of pro-inflammatory factors | Enhance inflammatory response and pulmonary injury | 12 |
|  | Release NETs | Via the TLR-4/TLR-9/NF-κB signaling pathway | Activate | Trigger activation of NLRP3 inflammasome and release of IL-1β | Enhance inflammatory response and pulmonary injury | 12 |
|  | Expression of hypoxia-inducible factor (HIF) | Expression under hypoxic conditions | Activate |  | Delayed resolution of inflammation and tissue damage | 12 |

**Supplementary Table 4** (continued)

| Virus type | Neutrophil Function | Interaction process | Neutrophil status | Active ingredient | Result | References |
| --- | --- | --- | --- | --- | --- | --- |
|  | Release ROS | The SARS-CoV-2 Omicron variant S protein and anti-spike protein IgG1 stimulate neutrophils to release myeloperoxidase (MPO), promoting the most reactive reactive oxygen species (ROS). | Activate |  | Exacerbate oxidative damage in lung tissue | 75 |
|  | Neutrophil serine proteases (NSPs) | Cutting viral S protein |  | Changes subsequent IFN-induced responses, such as high-level production of CXCL10 | Inhibits viruses, alleviates inflammation, and reduces lung damage | 77 |
|  | Increased expression of CXCR3 | The low-density inflammatory neutrophil population expressing moderate levels of CD16 exhibits pro-inflammatory gene signatures and activated platelets. |  | Form NETs | Promote phagocytic capacity | 74 |

**Supplementary Table 4** (continued)

| Virus type | Neutrophil Function | Interaction process | Neutrophil status | Active ingredient | Result | References |
| --- | --- | --- | --- | --- | --- | --- |
|  | Release ROS | The viral S protein and anti-spike protein IgG1 stimulate neutrophils to release myeloperoxidase (MPO), generating the most reactive reactive oxygen species (ROS). | Activate |  | Exacerbate oxidative damage in lung tissue | 75 |
|  | Increased levels of neutrophil granulase proteins such as DEFA3 and LCN2 | Neutrophil granulase can activate and enhance the complement cascade on NETs, which activate platelet and coagulation cascade reactions. | Activate | NETs | Severe condition | 80 |
| RSV | Decreased expression of neutrophil-activating protein NAP-2 and neutrophil-activating glycoprotein CD177 | Viral infection induces the strongest expression of interferon-related proteins (including ISG-15 and interferon λ1) and MCP-2 (a chemokine induced by interferon signaling), which shows a positive correlation with viral load. | Inhibit activation | Interferon type 1 | Impaired neutrophil response | 59 |

**Supplementary Table 4** (continued)

| Virus type | Neutrophil Function | Interaction process | Neutrophil status | Active ingredient | Result | References |
| --- | --- | --- | --- | --- | --- | --- |
| IV | Decreased expression of neutrophil-activating protein NAP-2 and neutrophil-activating glycoprotein CD177 | Viral infection promotes the strongest expression of interferon-related proteins (including ISG-15 and interferon λ1) and MCP-2 (a chemokine induced by interferon signaling), which shows a positive correlation with viral load. | Inhibit activation | Interferon type 1 | Impaired neutrophil response | 59 |
| IAV |  | IAV infection induces the establishment of a pro-inflammatory pulmonary environment via neutrophil infiltration, with neutrophils secreting mature and bioactive IL-1β. | Activate | IL-1β | Facilitates the establishment of a pro-inflammatory environment in the lungs | 125 |
| Airway inflammation | NLRP3 inflammatory small body; NETosis | Infection induces activation of NLRP3 inflammasome, NF-κB, and p38 signaling pathways |  | Maturation of IL-1β; Secretion of CCL3 and IL-6; Reactive oxygen species clusters | Increased inflammation | 68 |

**Supplementary Table 5** Comparison of dendritic cells with different viral infections

| Virus type | Dendritic cell function | Interaction process | DC cell state | Active ingredient | Result | References |
| --- | --- | --- | --- | --- | --- | --- |
| SARS-COV-2 | Pattern recognition receptors (PRRs) | The Toll-like receptor (TLR) family, including TLR1/2/6 or TLR3; retinoic acid-inducible gene I (RIG-I)-like proteins (RLRs, such as RIG-I, LGP2, and melanoma differentiation-associated protein 5); and nod-like receptors (NLRs, such as NLRC2 and NLRP3) recognize viral surface and intracellular receptors, activating the NF-κB and inflammasome pathways. | Activate | Stimulates pro-inflammatory factors such as IL-1β, IL-6, and TNF-α | Inhibit viral replication and limit viral transmission | 88, 90 |
|  | Generate type I interferon | Virus identification | pDC | Produces IFN-α | Restrict viral replication | 88 |
|  | TLR4 | Activation of TLR4 increases susceptibility of plasmacytoid dendritic cells to SARS-CoV-2 single-stranded RNA | pDC | Generates type I interferon | Inhibiting viruses | 92 |
|  | Express TLR7 | The reduction of IL-13 derived from T cells, while IL-13 can stimulate epithelial cell C-X-C motif ligand 12, thereby recruiting pDCs to the lungs during viral infection. | pDC | type I interferon | Inhibiting viruses | 88 |

**Supplementary Table 5** (continued)

| Virus type | Dendritic cell function | Interaction process | DC cell state | Active ingredient | Result | References |
| --- | --- | --- | --- | --- | --- | --- |
|  | CCR7 and CCL21 receptors | PGE2 secreted by MSCs inhibits DC maturation via interactions with CCR77 and CCL21, and also blocks monocyte differentiation into DCs by secreting soluble factors such as HIA-G, which inhibits the secretion of cytokines including TNF-α, L1-α, β, IL-6, IL-7, IL-8, IL-9, GM-CSF, and IFN-γ. | Inhibit | Cytokines such as TNF-α, iL-1β, and IL-6 | Exacerbate lung tissue damage | 38 |
|  | IL3 receptor | IL-3 stimulation of pDCs preferentially drives T cell differentiation into Th2 cells; induces IFN-λ expression | pDC | Synthesize IFN-γ | Resist viruses | 95 |
| RSV | Express TLR4/7 | Detection of the RSV F protein | Activate | Production of type I IFN; high levels of IFN-γ | Reduce inflammation and inhibit viral transmission | 88 |

**Supplementary Table 5** (continued)

| Virus type | Dendritic cell function | Interaction process | DC cell state | Active ingredient | Result | References |
| --- | --- | --- | --- | --- | --- | --- |
|  | Pattern recognition receptors (PRRs) | The Toll-like receptor (TLR) family, including TLR1/2/6 or TLR3; retinoic acid-inducible gene I (RIG-I)-like proteins (RLRs, such as RIG-I, LGP2, and melanoma differentiation-associated protein 5); and nod-like receptors (NLRs, such as NLRC2 and NLRP3) recognizes viral surface and intracellular receptors, activating the NF-κB and inflammasome pathways. | Activate | Induce pro-inflammatory factors such as IL-1β, IL-6, and TNF-α, as well as interferon | Inhibit viral replication and limit viral transmission | 88，100 |
| IV | Sensor | Identification of pathogen-associated molecular patterns (PAMPs) | Activate | Dangerous signals (including pro-inflammatory factors such as IL-1, TNFα, and IL-12, or anti-inflammatory factors such as IL-4, IL-10, and IFNγ) will be triggered. | Initiating widespread immune system activation through innate immunity | 86 |

**Supplementary Table 5** (continued)

| Virus type | Dendritic cell function | Interaction process | DC cell state | Active ingredient | Result | References |
| --- | --- | --- | --- | --- | --- | --- |
| IAV | Express CCR2 | During IAV infection, cDC progenitor cells (pre-cDCs) migrate to the lungs under the regulates of C motif ligand 2, resulting in increased production of cDCs. | Differentiate into cDC1s、cDC2s | Activate CD8 T cell responses; promote tissue-resident memory T cell responses | Remove virus | 88，94 |
|  |  |  | Differentiate into pDCs | Enhances the differentiation of plasma cells that secrete antibodies, thereby leading to antibody production | Remove virus | 88 |
|  | Pattern recognition receptors (PRRs) | The Toll-like receptor (TLR) family, including TLR1/2/6 or TLR3; retinoic acid-inducible gene I (RIG-I)-like proteins (RLRs, such as RIG-I, LGP2, and melanoma differentiation-associated protein 5); and nod-like receptors (NLRs, e. g NLRC2 and NLRP3) recognize viral surface and intracellular receptors, activating the NF-κB and inflammasome pathways. | Activate | Induces pro-inflammatory factors such as IL-1β, IL-6, and TNF-α | Inhibit viral replication and limit viral transmission | 88 |

**Supplementary Table 5** (continued)

| Virus type | Dendritic cell function | Interaction process | DC cell state | Active ingredient | Result | References |
| --- | --- | --- | --- | --- | --- | --- |
|  | TLRs | Recognition of endocytosed free viral particles by nuclear endosomal TLRs | pDC |  | Resistance to viral infections | 91 |
|  |  | The nonstructural protein 2 (NS2) of IAV binds to Xpo5, inhibiting the biosynthesis of miRNAs that impair antigen presentation; NS2 directly interacts with interferon regulatory factor 3 (IRF3), also inhibiting the antigen-presenting capacity of dendritic cells (DCs). | Suppress |  | Promoting viral spread | 104 |
| Vaccinia virus (VACV) | High-level nuclear lamina protein A/C | VACV influences dendritic cells (DCs) through TLR2/4, while DCs lacking nuclear envelope proteins A/C exhibit reduced ability to form immune synapses with CD4 T cells, leading to diminished activation, proliferation, and Th1 differentiation of CD4 T cells. | deletion | IFN-γ reduce | Reduced antiviral efficacy | 87 |
| Human cytomegalovirus (CMV) | Increases levels of IFA-1; TLR9 | Discriminate CMV | pDC、cDCs | Induce IFN-γ and/or other cytokines such as TNF-α | Control the virus | 91 |

**Supplementary Table 5** (continued)

| Virus type | Dendritic cell function | Interaction process | DC cell state | Active ingredient | Result | References |
| --- | --- | --- | --- | --- | --- | --- |
| EB virus | Stimulates TLR9 and MHC II expression | EBV DNA induces Th17 cell responses via the endosomal TLR signaling pathway to produce pro-inflammatory mediators IL-17A, which enhances the expression of MHC II molecules on dendritic cells (DCs). |  |  | Reduction of tissue inflammation | 93 |

**Supplementary Table** **6** Comparison of myeloid suppressor cells with different viral infections

| Virus type | MDSCs function | Interaction process | MDSCs state | Active ingredient | Result | References |
| --- | --- | --- | --- | --- | --- | --- |
| SARS-COV-2 |  | Downregulates of CD3ζ chain by Arg-1, iNOS, and TGF-β inhibits T cell proliferation and IFN-γ production | M-MDSCs | IFN-γ | Inhibition of viral clearance, leading to increased disease severity | 15,16 |
|  |  | IL-6 and IL-10 induce the expansion of M-MDSCs | M-MDSCs |  | Severe condition | 15 |
|  | Express NOX-1 and NOX-2 | Arg1+ G-MDSCs with overexpression of NOX-1 and NOX-2 reduce the expression of T cell receptor ζ chain and impair endothelial cell function, thus damaging T cell receptors. | G-MDSC | ROS | Promote patient mortality | 15 |
|  | Expressing PD ligand 1 and Fas ligand | Inflammatory factors such as IL-10 and TGF-β can activate MDSCs to produce PD-L1 and FasL, leading to T cell apoptosis and promoting T cell exhaustion. |  | IL-10、TGF-β | immunosuppressive | 17,107 |
|  | Express COX-2 | COX-2 expressed by MDSCs stimulates the generation of PGE2-mediated lymphopenia in COVID-19 patients | M-MDSCs | PGE2 | immunosuppressive | 107 |

**Supplementary Table** **6** (continued)

| Virus type | MDSCs function | Interaction process | MDSCs state | Active ingredient | Result | References |
| --- | --- | --- | --- | --- | --- | --- |
|  |  | The spike glycan of SARS-CoV-2 exhibits sialic acidification. The sialic acidified secretory glycoprotein (SGP) of SARS-CoV-2 binds to CD33-related Siglec receptors, subsequently leading to CD33-mediated MDSC activation via arginase 1, which impairs the immune responses of T cells and B cells. | CD33 MDSC amplification | Immunosuppressive cytokines such as TGF-β and IL-10 | Enhanced tissue damage | 15 |
| IAV | TLR7 | Viral RNA activates TLR7 to promote MDSC differentiation, significantly suppressing IAV-specific T-cell responses through the expression of Arg-1 and iNOS. | Widespread proliferation | Arg-1 and iNOS | Resulting in higher IAV titers and mortality rates | 15 |
|  | CD11b+GR1+ | Suppression of CD8+ T cells | hyperplasia | Arg-1 and iNOS | immunosuppressive | 18 |
| IV |  | Infection-induced MDSC expansion suppresses T lymphocyte and NK cell function by increasing IL-10 release, PD-L1 expression, and Arg-1 production. | hyperplasia | IL-10,PD-L1,Arg-1 | Stimulates persistent viral infection and damage to the host | 17 |

**Supplementary Table** **7** Comparison of T cells with different viral infections

| Virus type | T lymphocyte function | Interaction process | T lymphocyte status | Active ingredient | Result | References |
| --- | --- | --- | --- | --- | --- | --- |
| SARS-COV-2 | High expression of CD38, CD69, or PD-1 | SARS-CoV-2-specific CD8 T cells exhibit high levels of effector molecules | Specific CD8 T cells | IFN-γ,TNF and CD107a | Prevention of severe cases | 118 |
|  | Express CD45RA,CD127,TCF-1 and BCL-2 | SARS-CoV-2 infection induces the formation of memory precursor CD8 T cells, which are associated with maintaining CD8 T cell responses and producing robust, durable CD8 T cell memory pools. | Specific CD8 T cells | IFN-γ,TNF and CD107a | Rapid virus clearance and reduction of disease severity | 118 |
|  | pp6 protein | The pp6 protein, as a major positive regulator of FoxP3, enables regulatory T cells lacking FoxP3 to more readily acquire a pathogenic phenotype characterized by pro-inflammatory cytokine expression under certain inflammatory conditions. | iTreg | pp6 protein | Prevention and treatment of immune imbalance | 115 |
|  | PD-1、TIM-3 | The virus stimulates T cells to transform into exhausted phenotype T cells. | Activation and depletion | IL-6 | Suppressing host immunity | 116 |

**Supplementary Table** **7** (continued)

| Virus type | T lymphocyte function | Interaction process | T lymphocyte status | Active ingredient | Result | References |
| --- | --- | --- | --- | --- | --- | --- |
|  | IL-18 receptor | After infection, macrophages release IL-18, which binds to IL-18 receptors (IL-18Rα and IL-18Rβ) on T cell membranes, forming heterodimeric complexes to propagate intracellular MyD88 signaling. This enhances the transcription of inflammatory genes by activating NF-κB. | Activate | IL-18 | pro-inflammatory | 127 |
|  | Fas receptor | When stimulated by inflammation, MSCs express Fas ligand which binds to the Fas receptor on T cells, leading to apoptosis. | Apoptosis | IFN-γ | Increased inflammation | 38 |
|  |  | The formation of prostaglandin E2 (PGE2), indoleamine-2,3-dioxygenase (IDO), transforming growth factor-β (TGF-β), and hepatocyte growth factor (HGF) effectively inhibits T cell proliferation. | Suppresses proliferation | PGE2,IDO,TGF-βand HGF | Inhibition of T-cell immune characteristics | 38 |

**Supplementary Table** **7** (continued)

| Virus type | T lymphocyte function | Interaction process | T lymphocyte status | Active ingredient | Result | References |
| --- | --- | --- | --- | --- | --- | --- |
|  | Retinoic acid-inducible gene-I (RIG-I)-like receptors (RLRs), toll-like receptors (TLRs), nod-like receptors (NLRs), and cyclic GMP-AMP synthase (cGAS) | SARS-CoV-2 may directly activate NLRP3 inflammasomes, thereby production endogenous adjuvant activity (provided by pyroptosis regulated by the nod-like receptor family and NLRP3 inflammasome activation), which initiates an appropriate adaptive immune response against the virus. | Activate | NLRP3 inflammatory small body | Prevention of severe progression | 130 |
| Respiratory syncytial virus (RSV) | PD-1、LAG-3 | Influenza virus induces functional impairment of virus-specific pulmonary CDD8+ T cells via inhibitory receptors | Specific pulmonary CDD8+ T cells |  | Protective immunopathology | 123 |
|  | PD-1 | RSV stimulates PD-L1 expression in bronchial endothelial cells (ECs), thereby reducing the secretion of cytotoxic molecules by effector CD8 T cells. | Effector CD8 T cells | IL-2, IFN-γ, and granulase B | The virus survives within infected cells. | 124 |

**Supplementary Table** **7** (continued)

| Virus type | T lymphocyte function | Interaction process | T lymphocyte status | Active ingredient | Result | References |
| --- | --- | --- | --- | --- | --- | --- |
| IV | Express CD45RA、CD127、TCF-1 and BCL-2 | SARS-CoV-2 infection induces the formation of memory precursor CD8 T cells, which are associated with maintaining CD8 T cell responses and generating robust, durable CD8 T cell memory pools. | Specific CD8 T cells | IFN-γ,TNF and CD107a | Rapid virus clearance and reduction of disease severity | 118 |
|  | Areg | Following influenza virus infection, IL-18 stimulate the synthesis of Areg by Treg cells. | Tregs | Areg | Tissue repair | 121 |
|  | Express Prkaa1 and Prkaa2 | During viral pneumonia, AMPK regulates DNA methyltransferase 1 to maintain metabolic homeostasis and mitochondrial activity. | AMPKα1/α2Treg | AMPK | Protect the tissue from injury | 122 |
|  | PD-1、LAG-3 | Influenza virus stimulates functional impairment of virus-specific pulmonary CDD8+ T cells through inhibitory receptors | Specific pulmonary CDD8+ T cells |  | Protective immunopathology | 123 |

**Supplementary Table** **7** (continued)

| Virus type | T lymphocyte function | Interaction process | T lymphocyte status | Active ingredient | Result | References |
| --- | --- | --- | --- | --- | --- | --- |
|  | IL-18 receptor | After infection, macrophages release IL-18, which binds to IL-18 receptors (IL-18Rα and IL-18Rβ) on T cell membranes, forming heterodimeric complexes to propagate intracellular MyD88 signaling. This enhances the transcription of inflammatory genes by activating NF-κB. | Activate | IL-18 | pro-inflammatory | 127 |
| IAV | Express CD40 ligand | Auxiliary T cells express CD40 ligands that bind to CD40 on the surface of macrophages to potentiate their activation. | Helper lymphocyte T | IFN-γ,IL-4 | Promote and enhance the activity of innate and adaptive immune systems | 119 |
|  | Downregulation of IL-10 expression |  | Regulates CD4+ and CD8+ T cells | Inhibition of IL-10 | Exacerbate inflammation and tissue damage | 119 |

**Supplementary Table** **7** (continued)

| Virus type | T lymphocyte function | Interaction process | T lymphocyte status | Active ingredient | Result | References |
| --- | --- | --- | --- | --- | --- | --- |
|  | TCR | Cytotoxic T cells are activated by TCR and other co-stimulatory signals, producing perforin and proteases to form channels on the target cell membrane and degrade cellular proteins. Furthermore, apoptosis of target cells can be produced via the Fas-Fas ligand interaction. | CD8+T cell | Granulase, perforin, and production of immunomodulatory cytokines such as IFN-γ, TNF, and IL-10 | Virus clearance and mortality reduction | 119 |
|  | Express surface proteins CTLA4, lymphocyte activation gene 3, CD39, CD73, and CD25 | Induction of CD4+ T cell differentiation through transcription factor Foxp3 expression | Tregs | IL-10, IL-35, and transforming growth factor β | Sustaining immune homeostasis | 119 |
|  | Expressing αβTCR; TCR composed of γ and δ subunits | Recognition of conserved antigens on MHC-like molecules; capable of identifying conserved non-peptide antigens upregulated by cells under stress | NKT；γδT cell | IFN-γ；IL-17,IL-33 | Drive organizational protection and recovery | 119 |

**Supplementary Table** **7** (continued)

| Virus type | T lymphocyte function | Interaction process | T lymphocyte status | Active ingredient | Result | References |
| --- | --- | --- | --- | --- | --- | --- |
|  | Express IL-1R1 | CD8 T cells dynamically upregulate their responsiveness to IL-1 during the activation process of antigen-presenting cells, and memory CD8 T cells can maintain high levels of IL-1R1. | CD8 T cell | IL-21,IL-1,IL-1β | Inhibition of inflammation | 125 |
| CMV | Express CD45RA、CD127、TCF-1 and BCL-2 | SARS-CoV-2 infection induces the formation of memory precursor CD8 T cells, which are associated with maintaining CD8 T cell responses and generating robust, durable CD8 T cell memory pools. | Specific CD8 T cells | IFN-γ,TNF and CD107a | Rapid virus clearance and reduction of disease severity | 118 |
| EBV | Express CD45RA、CD127、TCF-1 and BCL-2 | SARS-CoV-2 infection induces the formation of memory precursor CD8 T cells, which are associated with maintaining CD8 T cell responses and generating robust, durable CD8 T cell memory pools. | Specific CD8 T cells | IFN-γ,TNF and CD107a | Rapid virus clearance and reduction of disease severity | 118 |

**Supplementary Table** **7** (continued)

| Virus type | T lymphocyte function | Interaction process | T lymphocyte status | Active ingredient | Result | References |
| --- | --- | --- | --- | --- | --- | --- |
|  | IL-18 receptor | After infection, macrophages release IL-18, which binds to IL-18 receptors (IL-18Rα and IL-18Rβ) on T cell membranes, creating heterodimeric complexes to propagate intracellular MyD88 signaling. This ultimately promotes the transcription of inflammatory genes by activating NF-κB. | Activate | IL-18 | pro-inflammatory | 127 |
| H9N2 | PD-L1 | The H9N2 virus suppresses T-cell immune responses by increasing PD-L1 expression in pulmonary microvascular endothelial cells. | Inhibit | Inhibition of IL-2, IFN-γ, as well as granulase B and perforin expression | Promote viral immune evasion | 124 |
| EB virus | Production of IL-17A | EBV stimulates Th17-mediated IL-17A production via the endosomal TLR signaling pathway | Th17 | IL-17A stimulates the expression of IL-6,IL-8,TNF-α,GM-CSF and MCP-1/2 | Enhance tissue inflammation | 93 |
